# Spectroscopically Evaluating Composition and Crystallinity in Pristine and UV-Exposed PET Films Using Polarization-Dependent Optical Photothermal Infrared Microscopy

**DOI:** 10.1021/acs.analchem.6c01918

**Published:** 2026-09-25

**Authors:** Danuta Liberda-Matyja, Hsiu-Chin Huang, Tae Joon Cho, Li-Piin Sung, John Pettibone

**Affiliations:** † Materials Measurement Science Division, 10833 National Institute of Standards and Technology, Gaithersburg, Maryland 20899, United States; ‡ Department of Chemistry and Biochemistry, University of Maryland, College Park, Maryland 20742, United States; § Materials and Structural Systems Division, Engineering Laboratory, 10833 National Institute of Standards and Technology, Gaithersburg, Maryland 20899, United States; ∥ Department of Chemistry, Krieger School of Arts and Sciences, Johns Hopkins University, Baltimore, Maryland 21218, United States

## Abstract

In this study, we used polarization-dependent photothermal infrared (OPTIR) microscopy to extend infrared (IR)-based methods to characterize structurally and chemically modified polymer films. Because of the reported relationship with polymer crystallinity and stability, methods capable of monitoring and estimating changes in crystalline regions are paramount for understanding the performance and fate. To develop PTIR methods that could advance the understanding of plastic and polymer degradation, we examined UV-irradiated polyethylene terephthalate (PET) films using simulated photodegradation via a high-energy radiant exposure (SPHERE) weathering device. The well-studied UV-irradiated PET system provided a model to expand the IR characterization capabilities. Previously reported IR methods for determining crystallinity in bulk PET through glycol conformer fractions were combined with advanced structural determination methods to characterize thick and opaque cross-sectioned films using azimuthal angles and Hermans in-plane orientation functions, which extended the sample types available for analysis. To demonstrate the opportunity to decouple signatures from chemical and structural modifications in changing and chemically heterogeneous films, heterogeneity mapping was reported as a function of depth in cross-sectioned films using two-trace two-dimensional (2T2D) correlation analysis coupled with K-means clustering. Faster imaging procedures were also developed to limit possible effects from sample degradation and instrument drift during the acquisition.

## Graphical abstract

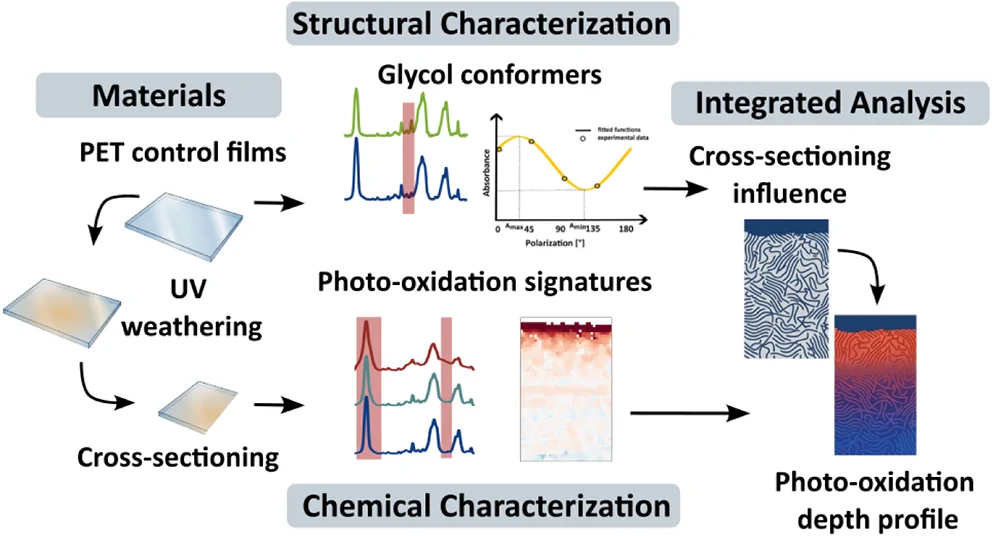

Mature bulk characterization and ensemble measurement methods exist for examining polymers and plastics under applied stresses, which are used to correlate changing material properties with changes to structure and composition (e.g., molecular weight). More recent evidence in laboratory-simulated weathering conditions has suggested that plastic particle generation predominantly originates from highly crystalline regions.^1,2^ However, the ability to differentiate structural and chemical degradation products resulting from physical and chemical stressors remains a persistent analytical challenge.^2,3^ Thus, conclusions about the mechanistic details of material degradation and particle generation have to be inferred rather than directly investigated, which has hindered material design and limited predictive models for material fate. Implementing higher spatial resolution microscopy capabilities alongside mature methods should promote progress toward elucidating more insight into specific chemical degradation, structural changes, and mass loss that affect material performance.

Our previous efforts to develop more sensitive methods focused on the design of reproducibly UV-exposed polyethylene terephthalate (PET) materials that could be used as a model weathered system for both film degradation and micro- and nanoplastic (MNP) particle sources.^4^ Semicrystalline PET materials are commonly used in commercial applications.^5^ There are three main phases—amorphous, intermediate-oriented amorphous, and fully crystalline—each exhibiting unique vibrational signatures. Previous work that has been reproduced for multiple PET materials has established that the ratio of ethylene glycol moieties in the trans and gauche conformers can directly estimate phase and percent crystallinity in films.^6,7^ Thus, through controlled degradation, PET can serve as a model for developing correlative approaches to examine both changing chemical composition and structural stability.

Complementary to Raman spectroscopy,^8,9^ infrared (IR)-based methods have been used to resolve and map crystalline structure within films.^2,10,11^ Most of the reported work has been conducted using attenuated total reflectance Fourier transform infrared (ATR-FTIR) spectroscopy. While reflection and ATR modes can be employed to characterize PET crystallinity, previous measurements were mostly performed by collecting single spectra and with limited depth resolution.^2,12,13^ These spatial limitations obscure fine, heterogeneous structures within the films. IR-based microscopy methods to improve spatial- and depth-resolved measurements of polymeric films need to be applicable to opaque materials^14^ and overcome limitations from signal saturation that occur with IR transmission measurements, which has been reported for PET films ≥50 μm.^15^ Emerging optical and vibrational spectroscopy methods are being developed to address the sensitivity and resolution requirements.^16–20^

Midinfrared photothermal microscopy, also known as photothermal infrared (PTIR) microscopy, is a group of high-resolution techniques that provide improved spatial resolution and limits of detection that can be used to investigate chemical and structural changes at relevant length scales for polymer systems. Optical photothermal infrared (OPTIR) microscopy, which is a class of PTIR techniques, has been commercialized and uses a visible laser as the probe to detect absorption from a tunable IR laser across the mid-IR spectral range. Using the optical probe enables submicrometer resolution capabilities with improved detection limits compared to conventional IR methods^21–24^ and provides orthogonal techniques for examining different MNP size volumes.^19,25,26^ OPTIR methods have been used to examine degraded films that result in particle generation, where single-particle spectroscopy was demonstrated.^26,27^ To build upon developed characterization methods for polymer-based systems, efforts to transfer and expand IR-based methods to OPTIR are necessary to fully exploit the enhanced capabilities.

The implementation of polarization-dependent OPTIR methods is an example where modification or extension of previously developed IR-based methods can provide new insight into both bond-specific and structural changes occurring at submicrometer lengths. Polarization measurements can be employed to determine polymer molecular chain orientation in two- and three-dimensional space by evaluating changes in transition-dipole moments parallel and perpendicular to the main molecular chain axis.^28,29^ In ideal anisotropic samples, the highest absorbance of linearly polarized IR light occurs when the transition dipole moments change parallel to the polarization direction while perpendicular polarization is not absorbed. This linear dichroic behavior was used to characterize mechanically stretched PET samples, providing insight into PET deformation through polymer chain orientation.^11,30–32^ Polarization-dependent methods have also been used to map molecular orientation in a water bottle based on two perpendicular polarizations of IR light.^33^ A more complete picture can be developed with more advanced data analysis tools. With the four-polarizations (4p) technique, a complete two-dimensional map was constructed for crystalline poly-ε-caprolactone (PCL) films using Hermans in-plane orientation function and azimuthal angle, which can be extended to calculate three-dimensional molecular orientation and order parameters using the orthogonal-pair polarization IR (OPPIR) analysis.^28,29,34^ Further work on crystalline PCL films with OPTIR demonstrated high-resolution orientation mapping of the lamellae structures that was validated by direct comparison with IR and Raman results.^29^ These studies also demonstrated the interoperability of IR-based methods to improve the data quality necessary for predictive models of relevant commercial plastics.^35^

The development of spectroscopy-based methods to isolate contributions from chemical changes would benefit from reproducible processing procedures. UV irradiation and other simulated weathering processes introduce additional spectral complexity from changing chemical moieties and molecular chain rearrangement in the exposed regions. The depth-dependent evaluation of UV-exposed films have been previously reported, and the regions where chemical modifications are most pronounced were generally less than 5 μm from the surface.^8,36^ However, to examine depth-dependent changes, cross-sectioning or other sample preparation procedures are required, which can introduce perturbations to sample structure. Additional sources of uncertainty when estimating crystallinity in polymer films have also been reported.^4^ An improved ability to monitor chemical-specific modifications leading to fracture and mass loss should improve understanding of structure–property relationships for weathered films. However, although OPTIR methods are rapidly advancing in polymer and plastic characterization, additional efforts to understand signal contribution from changing thermal properties in samples, sampling-depth effects, and measurement artifacts have not beenfully developed.^37–41^ Consequently, standardized sample measurement procedures and data processing workflows are still needed.

In this work, we applied submicrometer, polarization-dependent OPTIR microscopy to differentiate spectral changes associated with chemical transformation, molecular structure, and sample processing after UV irradiation. Because optimized OPTIR measurement methods to characterize pristine and weathered PET samples have not yet been developed, the systematic measurement approach used in the current work is outlined (Figure 1). Color-coordinated pathways were included to illustrate measurement method approaches used for each sample type, where green arrows represent molecular orientation and crystallinity evaluation, and blue arrows represent depth-dependent chemical mapping. Further details for how each sample was used and method the implementated were included in the figure caption and Materials and Methods section.

**Figure 1:**
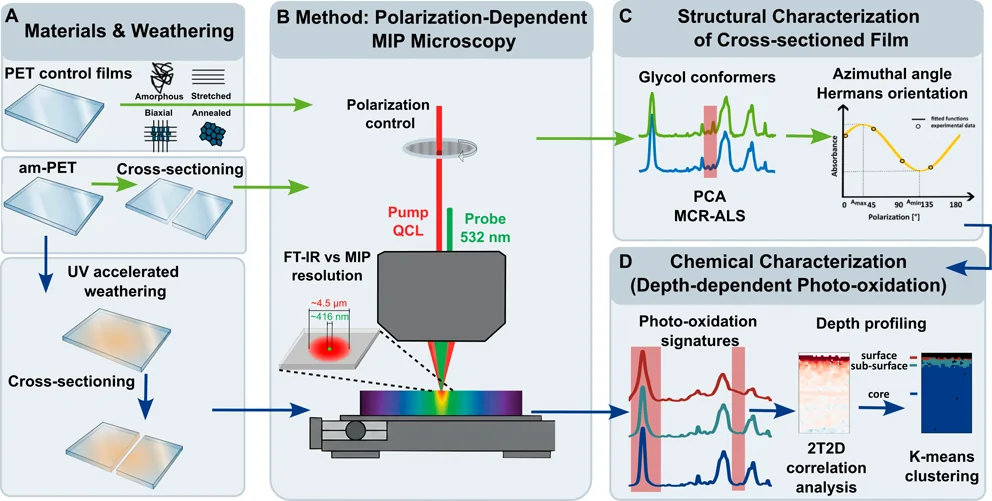
Experimental workflow: (A) material types, sample processing procedures, and simulated weathering approaches are presented, (B) illustration of OPTIR microscopy measurements and comparison of spatial resolution for theoretical FT-IR and OPTIR at 1725 cm^–1^ (inset), (C) summary of tools for evaluating structural characterization of cross-sectioned films, (D) method summary for evaluating photooxidation products characterized as a function of depth. The arrows represent the workflow and methods implemented for characterizing the PET samples: Green arrows: sample preparation using blade and microtome cross-sectioning to evaluate perturbations to molecular orientation and crystallinity using polarization-dependent OPTIR; Blue arrows: depth-dependent characterization of UV-weathered PET films measured with a single polarization angle −0°.

## Materials and Methods

### Reagents and Materials

*Control samples.* Five PET control film samples were purchased or prepared for characterization that represented a range of crystalline structures, which included amorphous (am-PET), two mesomorphic samples (amorphous PET stretched at different draw ratios), annealed, and biaxially oriented crystalline. 1-mm-thick PET amorphous and biaxial films were purchased from Goodfellow (Coraopolis, PA). Mesomorphic PET samples were prepared by stretching the pristine am-PET films using an RSA-G2 Solids Analyzer (TA Instruments, New Castle, DE, USA)[Fn fn1] in a tensile mode. The stretching process was performed at 80 °C, with 10 min thermal equilibration before stretching, and was conducted in two stages to obtain samples with different elongation levels. In the first stage, the am-PET films were uniaxially stretched to 40% and 80% elongation, designated as “stretched 1” and “stretched 2,” respectively. In the second stage, these samples were subjected to an additional stretching step using the same elongation ratios. In both stages, the initial gauge length was set at 15 mm, and the stretching rate was maintained at 0.1 mm/s.

The annealed film was prepared in the laboratory oven using a modified method.^42^ The am-PET sample was placed in the oven at room temperature and heated gradually (approximately 1 °C/min) to 165 °C, held for 4 h, and then gradually cooled to room temperature. Annealing am-PET resulted in a visual change from transparent to off-white and opaque.

### SPHERE Aging Experiments

Accelerated weathering was conducted in the NIST 2 m SPHERE (Simulated Photodegradation via High Energy Radiant Exposure), where the incident UV wavelength range was (295–400) nm.^43^ The pristine am-PET films were exposed at 50 °C under a dry environment for 35 days (35 d).

### Sample Cross-Sectioning

Blade-cut cross-sectioning was performed using a steel single-edge razor on two different am-PET slices (designated as blade 1 and blade 2) to investigate sample preparation method reproducibility. Cross-sectioning was conducted using a microtome (Leica Biosystems Nussloch GmbH, Nussloch, Germany). A glass knife was used to continuously flatten the cross sections, and the specimen feed increment was set to 1 μm per step. After each advance, the knife was cleaned to prevent contamination and/or surface damage that could affect the quality of the cutting surface.

### OPTIR Measurements

The photothermal microscopy measurements were conducted using a commercial optical photothermal infrared (OPTIR) mIRage-LS system (Photothermal Spectroscopy Corp., Santa Barbara, CA) in the copropagating mode with a 40× Cassegrain objective (0.78 NA). The MIRcat quantum cascade laser (QCL) and a 532 nm laser were used as the pump and probe beams, respectively. The polarized QCL laser was tuned to a 100 kHz pulse rate and 8% power, while the 532 nm laser power was set to 2.8% with a 20× standard detector gain. To control polarization of IR light, a wire grid polarizer was used. We defined light polarized parallel to the intrinsic QCL laser polarization direction as 0° (Figure 2C). Stretched and cross-sectioned films were placed under the microscope with the principal symmetry axis, defined as the main molecular chains oriented according to the stretch direction, aligned at 90°. Polarizer rotation away from 0° diminishes IR power intensity by a factor of cos^2^θ, where θ is the angle between the incident light direction and the polarizer transmission axis, according to Malus’s law. Therefore, molecular orientation and nonpolarized spectra approximation were calculated based on five different incident radiation polarization angles (5p), which included 0°, 45°, 60°, 120°, and 135°. Prior to background collection, pump and probe beam alignment was optimized for each polarization separately on the same low-e substrate. The respective optimized IR beam position coordinates and background were later uploaded before sample measurement. The polarization angle was tuned for a given sample by rotating the wire grid polarizer before collection of all spectra or images, starting with polarization 0°.

**Figure 2:**
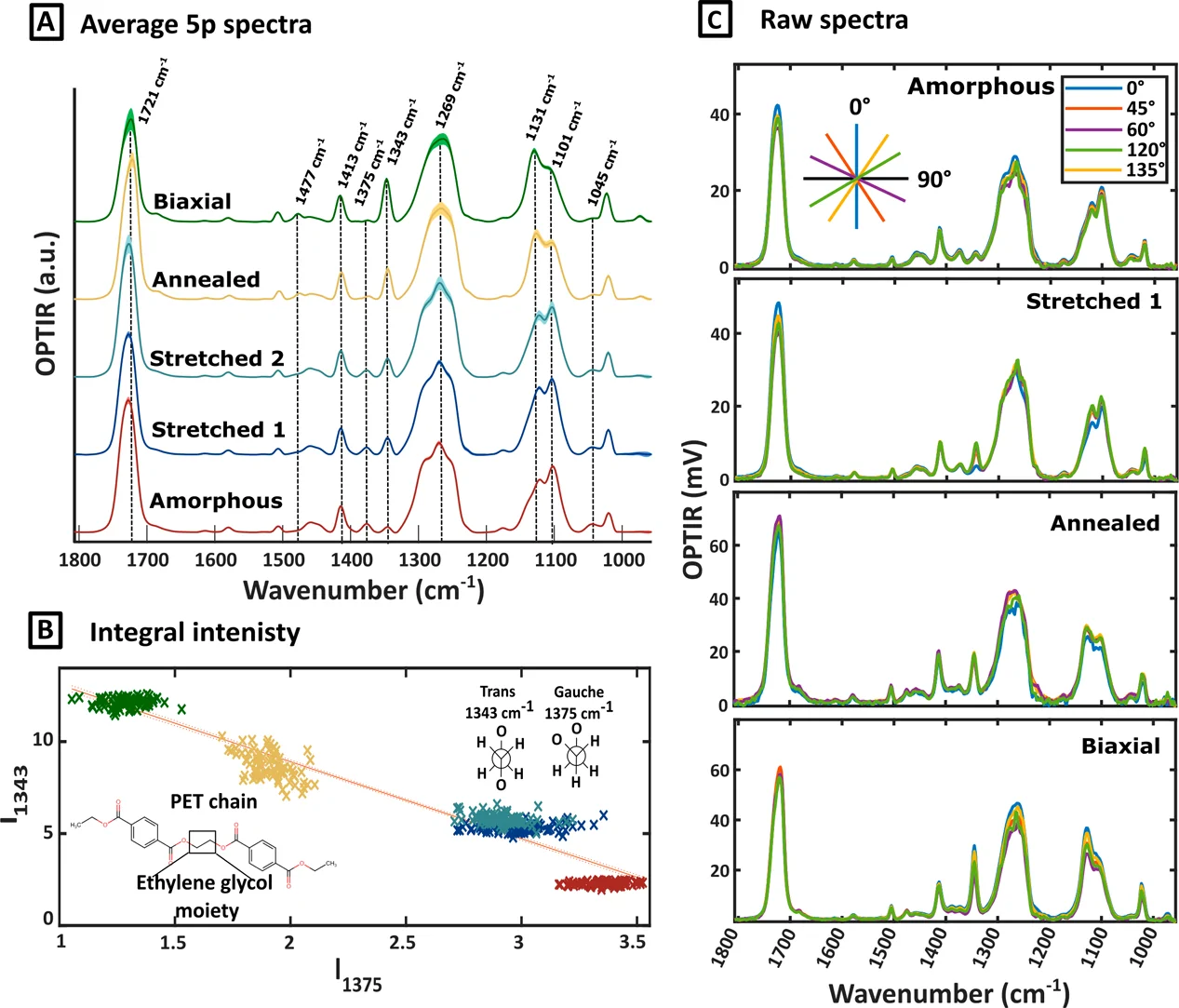
OPTIR spectra for PET control samples measured with five different incident IR polarization angles (0°, 45°, 60°, 120°, and 135°): amorphous (red), stretched 1 (dark blue), stretched 2 (light blue), annealed (yellow), and biaxial (green). (A) Representative spectra averaged over all polarization angles, (B) integrated intensity plot for 1343 cm^–1^ (trans conformer) vs 1375 cm^–1^ (gauche conformer) bands and the linear regression fit (five polarizations measured in 80 locations for each sample; *R* ^2^ = 0.938, *p* < 0.001), (C) raw polarized spectra measured from the same sample location at each polarization angle. The 0° polarization angle was referenced to the IR laser polarization. The measurement locations on each sample were presented (Figure S4).

Experimental parameters and acquisition settings for all OPTIR measurements described are summarized in Table S1 in SI. For control film samples, 100 spectra per sample were measured. 16 and 4 scans were collected for each background and sample, respectively. The OPTIR signal-based autofocusing was used to measure spectra in 1807–957 cm^–1^ range with 6.6 cm^–1^ spectral resolution. Cross-sectioned am-PET samples were imaged in 10 μm × 10 μm. Imaging was done with 200 nm step and 80 pt/s scan rate for 1343 cm^–1^, 1373 cm^–1^, 1413 cm^–1^ and 1331 cm^–1^, 1363 cm^–1^, 1391 cm^–1^, and 1431 cm^–1^ wavenumbers chosen as significant chemical contrast and baseline correction points, respectively. In imaging mode, focusing was done by minimizing probe beam spot size on the sample surface.

Hyperspectral measurements on the cross-sectioned weathered film sample were collected with 1 μm × 1 μm step (39 μm × 59 μm) using only 0° polarization in the horizontal and vertical directions, respectively, the same laser settings and number of scans were used for direct comparison to the unexposed control samples. Focusing was done by minimizing probe beam spot size on the sample surface.

To validate 0° polarization (0p) measurement results, a comparative 2T2D analysis was performed between the average of five polarizations including single 0° polarization of the same weathered sample. The measurements were collected in a new sample place using a 1 μm × 0.5 μm step in the horizontal and vertical directions over a smaller range of 17 μm × 23 μm, due to changing signal intensities during the long acquisition times required. Due to 80% longer five polarizations measurement time than in the case of only single polarization, QCL laser power was diminished to ≤4% to prevent sample damage.

For evaluating measurement reproducibility between samples and day-to-day performance, stability testing for both sample measurements and background collection was done daily to assess uncertainty from misalignment, drift, and any differences in the sample compartment environment. Assessments were qualitatively made by comparing relative peak intensity ratios for at least (*n* > 6) sample spots.

### ATR-FTIR Measurements

PET control films measured with OPTIR were also characterized using attenuated total reflection Fourier transform infrared spectroscopy (ATR-FTIR) (Thermo Scientific, Nicolet iS50, Waltham, MA, USA) equipped with a globar source. The nonpolarized ATR-FTIR spectra were acquired from 4000 cm^–1^ to 400 cm^–1^, with 4 cm^–1^ spectral resolution, averaged over 128 scans, and ATR wavelength-dependent corrections were applied in the OMNIC software. Measurements were performed at three different locations on the samples, 15 total spectra, to assess heterogeneity.

### Data Preprocessing

Data preprocessing of pristine film sample spectra was done by denoising using Savitzky–Golay (polynomial order 3, frame length 9), baseline correction with linear rubber bands (points: 1805 cm^–1^, 1775 cm^–1^, 1647 cm^–1^, 1621 cm^–1^, 1595 cm^–1^, 1559 cm^–1^, 1515 cm^–1^, 1493 cm^–1^, 1429 cm^–1^, 1389 cm^–1^, 1361 cm^–1^, 1329 cm^–1^, 1191 cm^–1^, 1063 cm^–1^, 1005 cm^–1^, and 955 cm^–1^), and normalization to 1413 cm^–1^ band maximum intensity. Normalization to 1410 cm^–1^ band was described before as introducing significant error being used as a thickness standard due to its positive dichroism, especially when analyzing polarized spectra (it can overestimate thickness by 18%).^31^ Calculation of structural absorbance, *A*_0_, has been proposed when dealing with uniaxial symmetry samples, assuming that absorbances for polarizations orthogonal to the stretching direction are identical $\left(A_{0}=\left(A_{∥}+2A_{⊥}\right)/3\right)$ .^31,44^ However, this approach was deemed inappropriate for samples with multiaxial symmetry. Therefore, normalization was performed before Hermans orientation function and azimuthal angle calculation due to signal intensity change between measured polarizations. Drift could occur during measurements, which were approximately 14 min long for each polarization. Beam positions may deviate between measured polarization directions depending on the stability of laboratory environmental conditions, and additional measurements were made to ensure reproducibility. Measured polarized spectra were averaged to analyze crystallinity signatures and calculate conformer fractions. Spectra with significant distortions of baseline and signal intensity were removed before analysis, based on the threshold values set for bands where signal intensity was significantly scaled. Model spectra represented an average of 80 measurements for each sample.

For the data collected in imaging mode, we corrected the baseline using the rubber-band method prior to calculating Herman’s orientation function and azimuthal angles. Due to relatively fast measurement times for imaging, signal scaling was not observed; thus, normalization was not applied. To calculate conformers’ fraction images collected with different polarization angles were averaged and normalized to 1413 cm^–1^.

Hyperspectral data collected for the UV-exposed sample were denoised using Savitzky–Golay (polynomial order 3, frame length 9), baseline-corrected using linear rubber bands (points: 1805 cm^–1^, 1647 cm^–1^, 1621 cm^–1^, 1595 cm^–1^, 1559 cm^–1^, 1515 cm^–1^, 1493 cm^–1^, and 955 cm^–1^), and normalized to 1413 cm^–1^ band maximum intensity. Analysis of outliers was consistent with the method used for the PET controls at individual polarization angles.

FTIR-ATR spectra of control samples prior to analysis were baseline-corrected and normalized. Baseline correction was performed using a rubber-band algorithm with anchor points at 1805 cm^–1^, 1755 cm^–1^, 1647 cm^–1^, 1621 cm^–1^, 1595 cm^–1^, 1559 cm^–1^, 1515 cm^–1^, 1493 cm^–1^, 1426 cm^–1^, 1389 cm^–1^, 1361 cm^–1^, 1329 cm^–1^, 1191 cm^–1^, 1163 cm^–1^, 1063 cm^–1^, 1005 cm^–1^ and 955 cm^–1^ Each spectrum was then normalized to the aromatic ring band at 1409 cm^–1^. The preprocessing approach used was similar to OPTIR measurements, but the band shapes and peak position differences in ATR-FTIR spectra required different anchor points for optimization.

### Data Analysis

2T2D analysis and corresponding plots were generated by using Python 3.12. The remaining calculations were done using MATLAB 2024b software.

### Conformer Fraction Calculation

The relative ratio of ethylene glycol moiety trans, *T*, and gauche, *G*, conformers in PET films has been used to calculate crystallinity.^7,12,31,45,46^ The relative conformational populations and characteristic absorbance, *A*, peaks used were described through:

1 $$T=\frac{A_{1340}}{A_{1340}+k\times A_{1370}}$$

2 $$G=\frac{k\times A_{1370}}{A_{1340}+k\times A_{1370}}$$

where *k* corresponds to the absorptivity ratio. *k* was determined from the slope of the regression line fitted to the model data, where the integral absorbances (*A*_1340_, *A*_1340_) were measured and plotted for samples of different crystallinity. For the current OPTIR measurements, integrated peak intensities for 1343 cm^–1^ and 1375 cm^–1^ bands were used.

The conformer fraction (crystallinity) calibration was performed for both ATR-FTIR and OPTIR data sets using five PET control samples (amorphous, stretched-1, stretched-2, annealed, and biaxial). In each case, integrated band intensities were obtained from the baseline-corrected, normalized spectra by summing the absorbance over *T* and *G* conformer regions. Least-squares linear regression (MATLAB fitlm) was fitted to the integrated *T* and *G* intensities. For the ATR-FTIR data, intensities were summed over the *T* conformer window (1331– 1353 cm^–1^) and the *G* conformer window (1360 cm^–1^–1389 cm^–1^), and the regression was fit across all 15 individual spectra (three per each sample type). The model exhibited a linear relationship between the two conformer bands (*R* ^2^ = 0.982, RMSE = 1.61, *F* = 705, *p* = 1.04 × 10^–12^, *n* = 15), covarying across the PET crystallinity range. For the OPTIR data, 80 spectra per sample type were used, totaling 400 spectra. Similar to ATR, intensities were summed over the *T* conformer window (1331 cm^–1^ to 1363 cm^–1^) and *G* conformer window (1363 cm^–1^ to 1391 cm^–1^), and the regression was fit across all 400 spectra. The resulting fit similarly exhibited a linear relationship between the two conformer bands (*R* ^2^ = 0.938, RMSE = 0.836, *F* = 6.03 × 10^3^, *p* = 1.62 × 10^–242^, *n* = 400). The preprocessing for ATR-FTIR and OPTIR differed slightly due to observed shifts in the corresponding peak positions for *T* and *G*; thus, conformer integration windows were adjusted independently for each method.

Simple factor analysis was also applied before on the whole spectral region to characterize different conformer fractions in stretched and annealed PET films, differentiating three phases: amorphous, intermediate (amorphous oriented), and crystalline.^12^ Here, the MCR-ALS algorithm was used to decompose measured data into pure-component spectral profiles (*S* ^T^) and their concentrations (*C*). Calculations were performed using MCR-ALS GUI v2.5.^47^ MCR performs bilinear decomposition of the analyzed data (*D)* assuming that measured spectra are linear combinations of pure components. It is expressed by the equation:

3 $$D=CS^{T}+E$$

where *E* is the matrix containing variance not explained by the model.^48^ In the MCR-ALS, this equation is solved by *C* and *S* ^T^ iterative optimization using alternating least-squares (ALS) approach under constraints. Here, MCR-ALS was utilized to find glycol moiety *G* and *T* conformers’ fraction (defining the number of components applied in the model as two) based on the full spectral region. Then, input data consisted of control and processed (blade and microtome-cut) sample spectra. Average amorphous and crystalline (annealed and biaxial) spectra were used as the initial standards. We applied nonnegativity constraint for both concentrations and spectral profiles. During algorithm optimization, normalization to keep spectra equal in height was applied. To express MCR-ALS fit quality, we calculated lack of fit and the percentage of explained variance.^48^

### Molecular Orientation

To characterize molecular orientation in analyzed film samples, we calculated azimuthal angle and Hermans orientation function using five polarizations (5p), similarly to the 4p method approach.^32,49^ Detailed description of the theory behind macromolecular orientation and Hermans orientation function was provided previously.^29,32,49–51^ With the 4p method to calculate azimuthal angle and dichroic ratio, absorbance at different polarization angles was measured to fit the nonlinear cosine function that relates absorbance *A*_γ_ and polarization direction:

4 $$A_{\gamma }=\frac{A_{\max}+A_{\min}}{2}+\frac{A_{\max}-A_{\min}}{2}\cos2\left(\gamma -\psi \right)$$

where *A*_max_ and *A*_min_ are maximum and minimum absorbance, *γ* is the polarizer angle, and *ψ* (azimuthal angle) is the polarizer angle when the absorbance is equal to *A*_max_. Four experimental points—a minimum of four polarizations (usually defined as 0°, 45°, 90°, and 135°)—are required to perform the fit. A higher number of polarization angles improves nonlinear function fitting results. In this study, the coordinate system for azimuthal angle calculation (Figure 4A) was defined with polarization 0° parallel to the incoming intrinsic QCL laser polarization direction. Based on the calculated nonlinear function, the dichroic ratio (for transition dipole moment parallel to the orientation axis) can be determined as

5 $$D_{\max}=\frac{A_{\max}}{A_{\min}}$$

which was used to calculate Hermans orientation function as follows:

6 $$f=\frac{D-1}{D+2}\cdot \frac{2}{3\cos^{2}\alpha -1}$$

where *α* is the angle between the main molecular chain axis and the transition dipole moment. In this study, we defined *α* angle as 18°, which was the previously reported approximation of CH_2_ wagging (1343 cm^–1^) transition dipole moment angle with respect to the PET molecular chain axis.^31^ Hermans orientation function achieves values equal to 1 for spectra or pixels of high molecular orientation parallel to the main molecular chain axis, isotropic character gives a value equal to 0, while −0.5 indicates high molecular orientation perpendicular to the main molecular chain axis.

### Two-Trace Two-Dimensional (2T2D) Correlation

Two-trace two-dimensional (2T2D) correlation spectroscopy^52,53^ is an analytical method where 2D cross-correlation synchronous **Φ** and asynchronous **Ψ** maps are calculated based on sample and standard (reference) spectra pair using the following formulas:

7 $$\Phi \left({ṽ}_{1},{ṽ}_{2}\right)=\frac{1}{2}[A_{1}\left({ṽ}_{1}\right)\cdot A_{1}\left({ṽ}_{2}\right)+A_{2}\left({ṽ}_{1}\right)\cdot A_{2}\left({ṽ}_{2}\right)$$

8 $$\Psi \left({ṽ}_{1},{ṽ}_{2}\right)=\frac{1}{2}[A_{1}\left({ṽ}_{1}\right)\cdot A_{2}\left({ṽ}_{2}\right)-A_{2}\left({ṽ}_{1}\right)\cdot A_{1}\left({ṽ}_{2}\right)$$

where ṽ_1_ and ṽ_2_ are a pair of wavenumbers to quantify the relative change in signal intensity between the sample *A*_1_ and reference *A*_2_ spectra. In the hyperspectral data set, each pixel spectrum was treated as the sample and compared against a single reference spectrum (the top surface of the amorphous, unexposed PET film) to map regions in which the chemical composition deviates from the unexposed state. **Ψ** > 0 values represent scenarios where species giving rise to ṽ_1_ are more prominent in the sample, *A*_1_, relative to the reference than the species giving rise to ṽ_2_, whereas **Ψ** < 0 indicates the opposite. The case when **Ψ** = 0 indicates that both ṽ_1_ and ṽ_2_ are synchronized and change proportionally between the sample and the reference (both bands arise from the same species, or neither has changed relative to the reference).

### Unsupervised Learning

K-means clustering was performed as an unsupervised learning method (using squared Euclidean distance metric for pixel assignment and fixed random seed) to find UV exposure signatures as a function of depth in cross-sectioned film based on the median-filtered (applying 3 × 3 pixels window) 2T2D Ψ values. The optimal number of clusters was defined as three, based on the elbow method, which minimizes the within-cluster sum of squares. The stability of the three-cluster framework was verified by calculating partition similarity across 10 independent random initializations.

## Results and Discussion

### PET Control Film Sample Crystallinity Characterization

The samples in Figure 1 represented commercially available PET films with increasing percent crystallinity. We stretched or annealed am-PET films to produce the intermediate “amorphous oriented” and crystalline structures, respectively. Because the IR laser sources are polarized in OPTIR systems, spectra for the control samples were averaged over five polarization angles to compare results collected from unpolarized light sources (Figure 2A). For each sample, the raw spectra of the five angles of incident light (0°, 45°, 60°, 120°, and 135°) are also reported (Figure 2C), where the color-coordinated legend for 0° collection angle was referenced to the polarization orientation of the laser. Expanded plots for the spectral region covering the two conformer bands were also presented for data clarity (Figure S1). The spectra for the PET film controls demonstrated bands consistent with previous absorbance IR spectroscopy studies.^35^ Thus, we were able to test previously reported absorbance methods developed for film samples and modify methods as necessary for samples with more chemical and structural heterogeneity (e.g., cross-sectioned films).

The characteristic bands for PET are marked with dashed lines to highlight spectral differences observed with changing molecular orientation and crystallinity (Figure 2A). We used a previously reported method developed with conventional IR using the glycol moiety *G* and *T* conformer fractions to estimate percent crystallinity (see methods eqs 1 and 2).^12,45^ In Figure 2B integrated peak intensities, *I*_*x*_, at 1343 cm^–1^ and 1375 cm^–1^ bands are plotted along the axes to evaluate conformer fractions, and therefore, crystallinity in the control samples . Based on the plot, *I*_1343_/*I*_1375_ increases monotonically from the am-PET (value = 0.68 ± 0.03), stretched 1 (*v* = 1.78 ± 0.10), stretched 2 (*v* = 1.99 ± 0.14), annealed (*v* = 4.56 ± 0.53) to biaxial crystalline sample (*v* = 9.44 ± 0.65). The linear regression fit for all data measured was represented by the red line (*R* ^2^ = 0.938). The absorptivity ratio was calculated from the slope of the regression line, *k* = 4.2. Stretched samples were clearly separated from the amorphous films based on the calculated *I*_1343_/*I*_1375_, and thus *T*/*G* composition (SI Table S2, Figure S2). The annealed sample had a lower *T/G* ratio than the biaxial sample, demonstrating the sensitivity of this method to distinguish crystalline structures with different molecular ordering. This methodology could be used for refining processing methods to tune material properties.^45,54^

Changes in the depth-dependent orientation can influence the measured *T/G* values for samples with anisotropic structures, such as the biaxial samples. For comparison, we measured the control film samples with attenuated total reflectance Fourier transform infrared (ATR-FTIR) spectroscopy and obtained results similar to those of the previous work, *k* = 6.6 (Figure S3).^45,46,55^ We noted that *k* is strongly dependent on the chosen data preprocessing strategy. Consequently, while the approximation of unpolarized light via the averaging of five polarization directions may have a subtle influence on the intensity response and differences between OPTIR and ATR-FTIR *k* values, we found that the discrepancies for *k* arise because the baseline parameters and integration regions had to be adjusted independently to correct for instrument- and method-specific artifacts. The linear fit across PET samples of varying crystallinities demonstrated performance of our preprocessing procedure. Because of the physical proximity of the *T* and *G* modes in PET, a minor spectral overlap is inherently present. Therefore, the derived *k* should be interpreted as an effective absorptivity ratio. The reported conformer fractions derived were intended as semiquantitative, method-specific descriptors rather than absolute crystallinity estimates.

Analysis of measured polarized spectra provided additional information about sample molecular orientation based on the previously reported bond angles approximated as parallel, ∥_1343_, and perpendicular, ⊥_1720_, to the main molecular chain at 1343 cm^–1^ and 1720 cm^–1^. Amorphous spectra did not exhibit polarization-dependent differences based on the ∥_1343_ and ⊥_1720_ ratio intensity (Figure 2C), consistent with a randomly oriented structure. “Stretched 1” sample exhibited induced orientation that followed the stretch direction described as 90° in our polarization measurement coordinate system (Figure 2C), where ∥_1343_ band had the lowest intensity for polarization 0° perpendicular to the stretch direction and maximum signal intensity observed in parallel at 60° and 120°. Opposite behavior was observed for ⊥_1720_ CO stretching vibration band centered between 1721 and 1725 cm^–1^. Bands 1269 cm^–1^, 1131 cm^–1^, and 1101 cm^–1^ also exhibited high polarization dependence, with a similar trend to parallel modes. In both annealed and biaxial samples, dichroic character of the spectra was observed. However, differences between signal intensity across measured polarizations were less prominent than for the stretched sample. Efforts to better model the depth-dependent signal origin for photothermal measurements are an active area of research,^38,39^ but are not the focus of the current study.

Other spectral signatures associated with structural changes in PET have also been previously reported, which were used for assessing the current method performance. Change in orientation of carbonyl groups in the terephthalate moiety with respect to benzene rings can be detected by examining the shift for CO stretching λ_max_ to lower wavenumbers and peak shape changes from Gaussian to more asymmetric as films become more ordered. The CH_2_ bending modes shifted from 1450 cm^–1^ to 1477 cm^–1^ with increasing crystallinity.^12^ Other work has examined ester group vibrations in the 1340–1060 cm^–1^ spectral region.^12,56^ Within the 1100–1120 cm^–1^ region, the intensity ratio of the crystalline and amorphous components has also been used to estimate crystallinity.^57,2,12^ Characteristic *G* and *T* conformer modes have also been previously assigned in the 1080–940 cm^–1^ region and used to calculate *T/G* composition.^58,59^ In general, these reported methods resulted in similar crystallinity estimates. However,these methods represent combination bands with more vibrational modes, which results in more complex peak decovolution and could introduce more uncertainty in calculated crystallinity.

### Sample Cross-Sectioning-Induced Molecular Orientation

To better understand the depth-dependent contributions of any mixed molecular orientations and sample preparation procedures, we cross-sectioned samples and evaluated spectral changes laterally from the surface and at increasing depths within the film. We used am-PET to evaluate induced order from the sample preparation procedures. Three film samples were cross-sectioned and evaluated, one prepared by microtome and two manually blade-cut. The spectra for the controls represented the average signal collected from the top surface of the films. To visualize similarities between cross-sectioned samples and model data, we applied principal component analysis (PCA) data exploration (Figure 3, SI Figure S5C). The loading plot identifies the spectral features responsible for sample separation observed in the score plot and thus explains the differences in chemical composition between the analyzed samples. Based on the scores plot (Figure 3A), am-PET and crystalline PET samples are separated primarily along PC2. The corresponding loadings (Figure 3B) showed that this separation was driven mainly by the 1739 cm^–1^, 1299 cm^–1^, 1459 cm^–1^, 1377 cm^–1^, 1097 cm^–1^, and 1045 cm^–1^ bands, which load negatively on PC2 and are characteristic of am-PET. Conversely, the more crystalline samples (biaxial and annealed), lying on the positive PC2 side of the scores plot (Figure 3A), were characterized by the bands loading positively on PC2: 1717 cm^–1^, 1347 cm^–1^, 1269 cm^–1^, 1131 cm^–1^ and 1025 cm^–1^. Spectra for the stretched samples that are mainly composed of the amorphous oriented phase were closer to am-PET than crystalline samples in the scores’ projection space (Figure 3A), which was consistent with the *I*_1343_/*I*_1375_ data that clearly differentiated biaxial and annealed samples. Also, PCA results based on spectra measured across multiple polarization angles, along with cumulative % of variance, resulted in a similar data distribution within the scatter plot (Figure S5).

**Figure 3:**
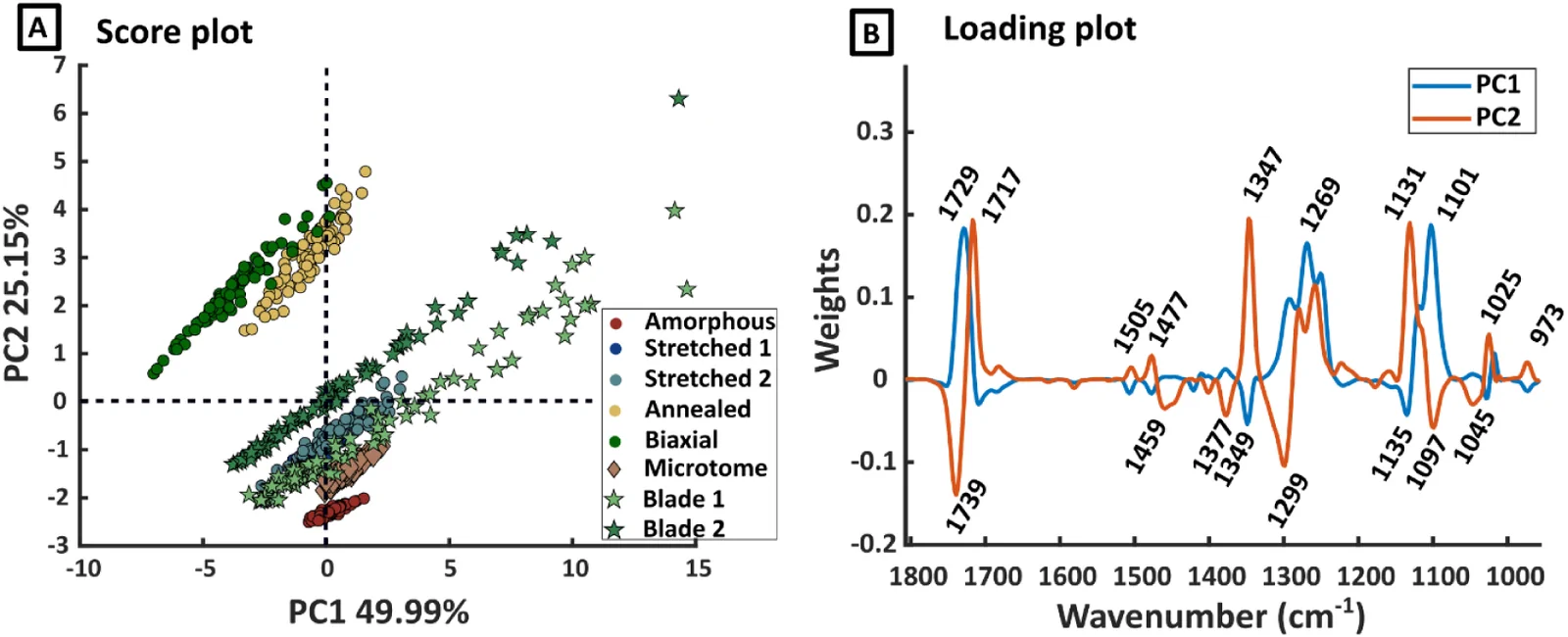
PCA results for PET samples and cross-sectioned am-PET films: (A) scores projection; (B) loading plot. Each point on the score plot represents one spectrum. A total of 80 spectra per sample were reported.

Microtome cross-sectioned sample spectra were located close to the am-PET in scores projection space and exhited lower variability across both PCs, which suggested less perturbed chemical composition. Blade-cut spectra corresponded most closely to stretched mesomorphic samples, indicating that the sample preparation method induced changes. Induced stretching or high molecular ordering (along the cut line) varied between independently prepared samples but also within the 10 μm × 10 μm area measured for each sample, respectively. To corroborate our findings, we calculated Hermans orientation function using 1343 cm^–1^ from the polarized OPTIR spectra and color-coded projected cross-sectioned samples in the PCA space (Figure S6A). Hermans orientation function for blade cross-sectioned samples had higher values than the microtome samples , indicating that sample preparation induced higher molecular orientation across the cut surface nonuniformly.

To remove possible interpretative bias resulting from examining individual features independently across samples, we applied the MCR-ALS algorithm to find conformer fractions based on full spectra (Figure S7). Both methods gave comparable conformer fraction trends for control data (Figure S8A and C). The *T* conformer fraction calculated with MCR-ALS was more uniform across cross-sectioned samples (color-coded projected in PCA score plot Figure S6B and C). Irrespective of the applied method, *T* conformer fractions were always lower for the microtome-prepared sample than blade-cut samples, which indicated that blade-cut samples introduced both conformational and structural changes (Figure S8).

This study represented a more complete characterization of molecular orientation in non-IR-transparent PET films than previously reported. The resulting data were possible by combining polarization-controlled OPTIR microscopy and advanced data analysis methods, including azimuthal angle determination (Figure 4A), Hermans orientation function (Figure 4B), and a validated conformer fractions method (Figure 4C). Combining these methods enables systematic evaluation of sample structure and resulting changes from sample production, processing, and preparation. Due to the time-intensive nature of high-resolution spectroscopic mapping, the sampling strategy was adjusted based on the expected structural impact of each cutting technique. Because microtoming induced less measured perturbation and resulted in a more uniform surface, analyzing one representative region was deemed sufficient. Conversely, because the blade method was expected to induce more significant and heterogeneous deformation, two independent specimens (blade 1 and blade 2) were analyzed to better capture the variance. The principal symmetry axis of each sample was defined along the stretching (chain-orientation) direction. In the measurement geometry, this axis was oriented at 90° with respect to the laser polarization at 0° (Figure 4A). Polarization-dependent OPTIR signal intensity changes were visible in the measured spectra (Figure 4D). Both samples had the lowest intensity for ∥_1343_ at polarization 0°, which was perpendicular to the sample cut direction. The highest intensities were observed at 60° and 120° for both samples. The polarization dependence for the samples being similarly oriented indicated that the direction of the cut influenced the ordering of the molecular chains.

**Figure 4:**
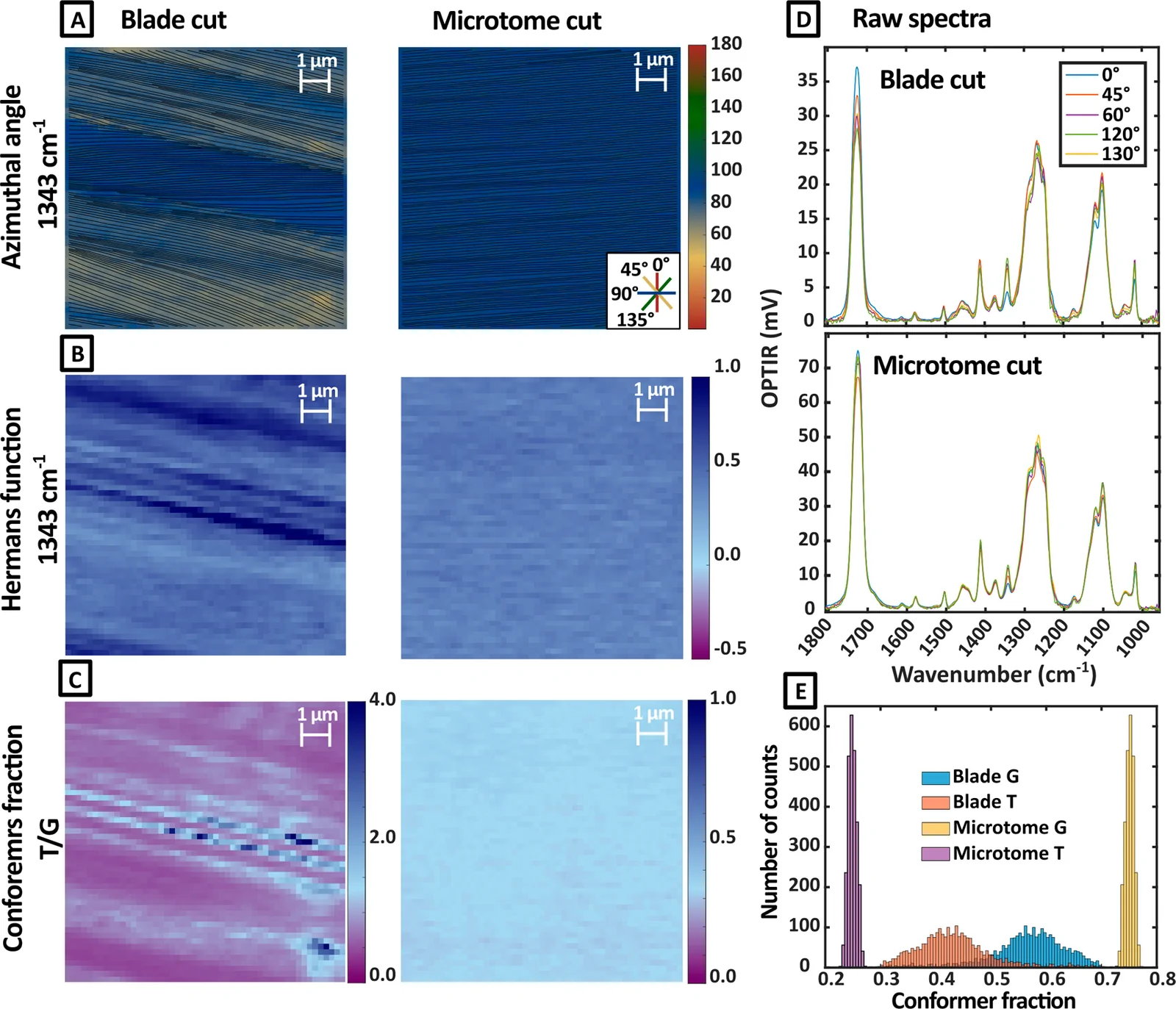
Blade and microtome sample cross-sectioning comparison based on: (A) azimuthal angle, (B) Hermans orientation function, (C) conformer fraction ratio, (D) single-point raw polarized OPTIR spectra, and (E) distribution of conformer fractions from images in panel C (51 × 51 pixels per image).

More details for the two-dimensional ordering space were presented by calculating azimuthal angle values (Figure 4A). Microtome samples had more uniform orientation in the inspected region, which exhibited molecular chain orientation following the 90° direction (blue). Blade-cut data exhibited induced molecular chain orientation in two directions that could be approximated as 60° (yellow) and 90° (blue). To demonstrate method performance, we also calculated azimuthal angles for the blade-cross-sectioned sample in the horizontal and vertical directions, which resulted in observed orientations at both 90° and 0°, respectively (Figure S9).

To quantify changes induced by the two sample preparation methods, we calculated Hermans orientation function for the cross-sectioned samples (Figure 4B). We used the median Hermans orientation function value obtained for am-PET equal to 0.1895 as the reference (Figure S10), where 0.0 would indicate isotropic character. Comparison of the Hermans orientation function with statistical significance between cross-sectioned and amorphous samples was also conducted (Figure S11). Calculated median Hermans orientation function values for processed samples were equal to 0.5387 and 0.4251 for the blade-cut and microtome samples, respectively. The values are consistent with azimuthal angle determination data for higher molecular ordering for blade-cut samples. Results for the “blade 2” sample can be found in SI (Figure S12). Although Hermans function values are helpful for visualizing structure, contributions from surface roughness that affect optimal OPTIR signal collection could introduce uncertainty in absolute molecular structure.

The conformer ratios were calculated for averaged polarized images using maximum peak intensities (reflecting nonpolarized images) and *k*, serving as a first-order approximation for a constant bandwidth ratio. This framework provides a comparative tool to track the relative spatial distribution and stress-induced evolution of the *T* and *G* conformers in the films. The calculated ratios of *T/G* and histograms for the distribution of individual conformers are presented (Figure 3). Histogram plots of calculated conformer fractions allowed visualization of the shift from *G* and *T* distributions prior to and after cross-sectioning. Initially, the am-PET film represented primarily *G* conformers (*G* > 0.8; Figure S10). The blade-cut samples demonstrated *G* to *T* conversion across measured areas and analyzed samples (Figure 4 and Figure S12–S13) with broad, nonuniform distributions. The differences demonstrated the large variation associated with the blade-cut samples. The distribution of *G* conformer fraction for the microtome sample fell between 0.72 < *G* < 0.78, which was consistent with Hermans function results that exhibited less sample perturbation. Cross-sectioning surface-induced orientation changes are the effect of cold drawing, where the film surface is stretched by a blade at a temperature below the PET glass transition. The difference between blade and microtome cut impact can be connected to applied blade type, applied force uniformity, and temperature in the laboratory environment, where microtome cut could be performed reproducibly. The application of both the conformer fraction and Hermans function values together provided visual inspection of the evolution of the sample handling that can be used to differentiate sample processing from other factors for more complex, weathered films.

### Photo-Oxidation Chemical Signatures in Cross-Sectioned Samples

After establishing structure-dependent PET signatures, we extended the method to deconvolute the chemical and structural effects that occur during UV irradiation. Hyperspectral measurements were performed on a microtomed sample. To compare OPTIR depth-resolved detection of photooxidation products with previously reported results,^60^ we applied a 2T2D method. Unlike a standard difference spectrum, the 2T2D asynchronous approach is immune from scaling issues and improves spectral resolution.^61^ This approach allowed deconvolution of heavily overlapped bands and the identification of distinct photooxidation products (specific peaks marked with dashed lines in Figure 5D) as a function of depth. These include ester derivatives at 1757 cm^–1^, benzoic acids end groups at 1697 cm^–1^, formation of carboxylic acid end-groups at 1429 cm^–1^ and broad, overlapping bands near 1205 cm^–1^ that are used to compare against the C–O stretching band centered near 1725 cm^–1^.^4^ The asynchronous, Ψ, and synchronous, **Φ**, 2T2D correlation contour maps containing identifiers for the chosen coordinates evaluated were presented (Figure S14). To optimize imaging throughput, a comparative 2T2D analysis was first performed between the average of 5p and single polarization, 0p, data set of the microtomed, 35 d UV-exposed PET sample. We observed identical features for 5p and 0p results (SI Figure S15 and S16 A–E), which demonstrated that a single polarization was sufficient to capture the same photo-oxidation gradient and chemical weathering boundaries while reducing instrument acquisition time by >80%.

**Figure 5:**
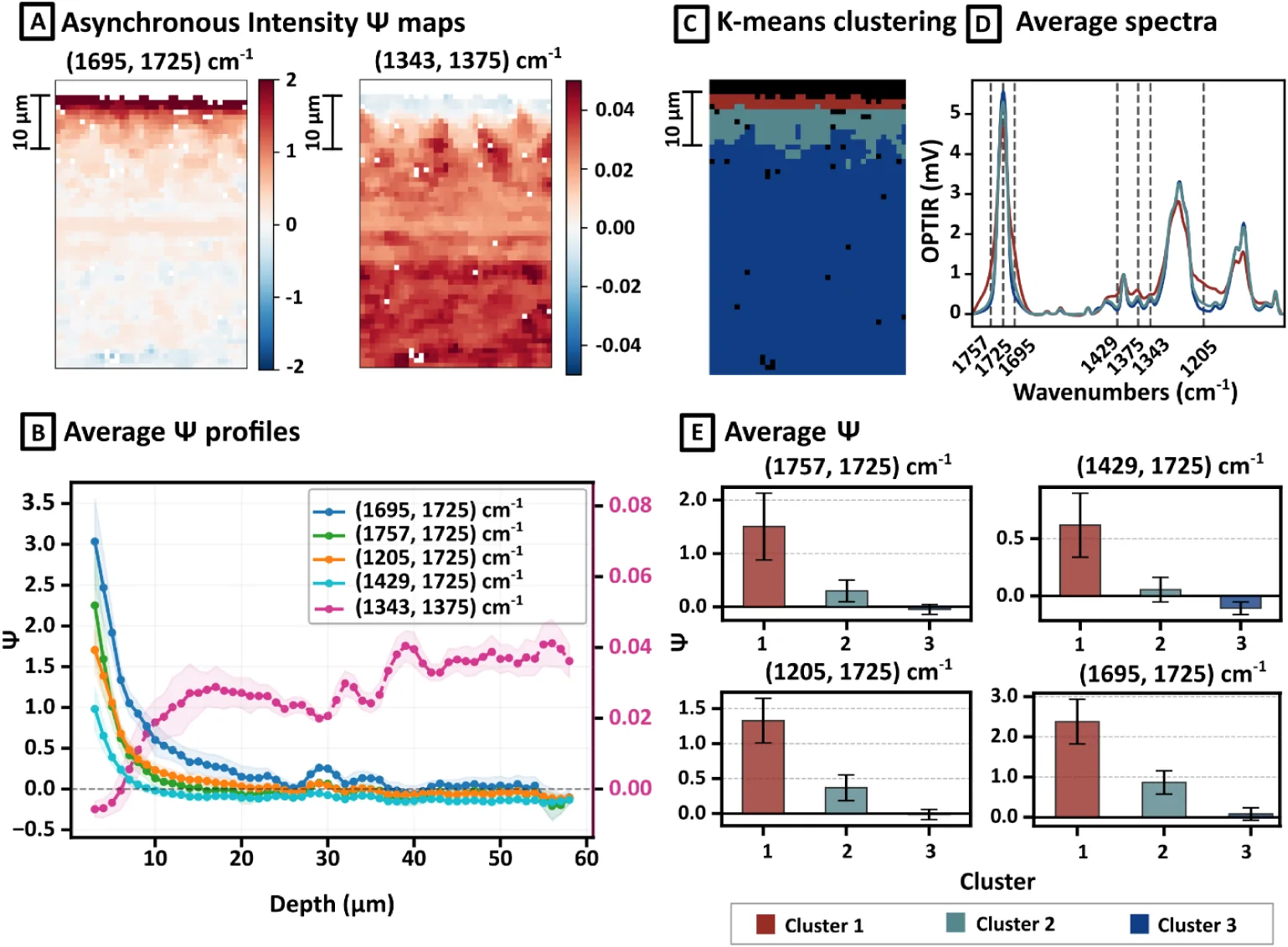
Spatial-depth and statistical evaluation of the 35 d UV-exposed am-PET film cross-section produced by microtome (2127 spectra after outlier removal): (A) spatial 2T2D asynchronous (Ψ) maps, (B) averaged 1D asynchronous depth profiles plotted as a function of distance from the UV-exposed surface (0 μm), (C) K-means clustering based on 2T2D results, (D) cluster average spectra with wavenumbers of interest, and (E) average asynchronous values for analyzed clusters with standard deviations.

Evaluation of the 35 d, UV-degraded am-PET, mapped at a 1 × 1 μm resolution extending 56 μm from the sample surface, was presented in Figure 5. In Figure 5A, the Ψ maps are shown for the degraded sample using comparisons between (1695 cm^–1^, 1725 cm^–1^) and (1343 cm^–1^, 1375 cm^–1^), which provide insight into chemical conversions and conformational changes associated with crystallinity, respectively. In the top ∼2 μm layer of the cross-sectioned sample at 1695 cm^–1^, 1725 cm^–1^, higher Ψ values are observed (Figure 5A), indicating that carboxyl species were enriched at the surface relative to the ester functionality when compared to the reference, unexposed film.^3^ The depth profiling provided a visual representation for the relative extent of ester bond cleavage that occurred, primarily in the top 10 μm. Additionally, growth of the benzoic acid peak (1695 cm^–1^) and decreasing intensity of the CO stretching (1725 cm^–1^) were consistent with the ester bond cleavage process. Although the penetration depth of the UV light is theoretically >30 μm, the appearance of the oxidized products was mainly at the film surface, which suggested secondary reactions are contributing to the conversion extent and consistent with our previous work.^4^ In the map of 1343 cm^–1^, 1375 cm^–1^, small negative Ψ values (0 to −0.02, light blue) close to zero were observed in the top ∼2 μm (light blue), indicating near-proportional behavior of the two conformer bands relative to the amorphous reference. Past ∼2 μm depth, Ψ values above zero were calculated, which indicated a change in the relative conformer fraction (Figure 5A). However, multiple issues could affect crystallinity estimates. First, samples were measured only with polarization 0° and could affect relative peak ratios, which was described in detail in the SI. Additionally, the photooxidation products resulted in broadening absorption bands that overlap with the bands used for estimating crystallinity. Therefore, only qualitative conclusions and estimates of changing crystallinity could be made. To directly obtain information regarding UV-induced crystallinity changes, both appropriate sample preparation evaluation and similar 5p measurement approaches should be implemented.

The average Ψ values as a function of depth for all analyzed wavenumber pair coordinates provided further insight into the method performance by comparing trends between wavenumber pairs (Figure 5B). Ψ values for the 1757 cm^–1^, 1697 cm^–1^, and 1429 cm^–1^ relative to 1725 cm^–1^ exhibited a decreasing trend within the first 10 μm, indicating a shift toward synchronicity and thus diminishing oxidation. Beyond 10 μm, near-synchronous Ψ values are observed, consistent with band ratios for unexposed am-PET. Conversely, 1343 cm^–1^ and 1375 cm^–1^ average Ψ values (pink trace, Figure 5B) were growing, following an asymptotic trend of the other pairs evaluated to the same 10 μm depth. Overall, the current method demonstrated that detecting and quantifying structural differences could be achievable, but additional research will be needed for accurate quantification.

2T2D analysis enabled the investigation of continuous changes as a function of depth. Here, applying K-means clustering on 2T2D results, we were able to provide discrete groups of photooxidation product distributions that were unique for different material regions (Figure S17). The surface (red), subsurface (light blue), and core (dark blue) cluster regions were identified in Figure 5C along with their corresponding averaged spectra (Figure 5D). Single-line scan spectra, as a function of depth up to 11 μm, were also presented for comparison (SI Figure S18). The dashed lines represented the analyzed wavenumber used for the combined analysis. This approach allowed us to define the depth of material degradation. The Ψ values could also be visualized as an average value within each cluster (Figure 5E). There were significant differences in Ψ values when comparing between clusters of 1757 cm^–1^, 1697 cm^–1^, and 1429 cm^–1^ against 1725 cm^–1^. A clear trend of reduced oxidation with increasing depth was observed, which resulted in the ability to differentiate the distinct regions: highly oxidized surface compositions (0–2 μm), subsurface population (3–10 μm) with moderate damage, and the least modified PET region (11–56 μm). Full asynchronous 2T2D IR correlation contour plots for each region were also calculated (Figure S19).

The current work validates the adaptation of analytical methods previously developed for traditional IR to PTIR spectroscopy methods, where improved characterization could be achieved for more polymer sample types. The methodology to integrate composition, molecular orientation, and changing crystalline structure for thick, opaque films can be implemented broadly to investigate more representative or other weathered materials. Additionally, because of the spectral fidelity and spatial resolution advantages of OPTIR over traditional IR measurements for increasing smaller analytes sizes, extension of the film characterization tools to sub-10 μm particle samples are readily accessible. Improved spatial resolution is likely a prerequisite for advancement toward mechanistic understanding of chemical and structural stability studies. Our previous work outlined efforts for characterizing UV-exposed cryomilled PET for the development of control materials.^4^ The current work represents an analytical advancement that should support both further method development and accelerate applied research efforts on detecting and characterizing both the forming secondary MNP populations and their point sources.

## Conclusions

This work provided the foundation to extend previously reported IR spectroscopy methods used for determining crystallinity and molecular orientation in transparent films to more complex samples. Polarization-dependent OPTIR microscopy measurements demonstrated perturbations to the PET chains that resulted from sample preparation, where molecular chain orientation and structural changes were detected and mapped. By implementing well-characterized controls and information on the contributions to sample surfaces from cross-sectioning, photo-oxidation signatures in cross-sectioned films could be distinguished by applying a combined 2T2D method. The results demonstrated that OPTIR can improve detection of photooxidation signatures in nontransparent cross-sectioned PET samples over conventional ATR-FTIR microscopy, while also resolving localized photooxidation products at or below the 1 μm scale. The 2T2D approach resolved individual photooxidation products and mapped their distribution with depth, revealing a surface-confined oxidation gradient in the top ∼10 μm. A direct comparison between single 0° polarization and five-polarization 2T2D analyses confirmed that a single 0° polarization is sufficient to capture the photooxidation chemistry. The two approaches diverged only for the conformation-sensitive bands; thus, quantification of UV-induced crystallinity likely requires more structure-sensitive approaches, such as the five-polarization method presented. Together, these results establish polarization-resolved OPTIR combined with 2T2D correlation as a promising tool for experimentally separating the chemical and structural contributions that result from weathering in opaque, semicrystalline polymers. More generally, this work provides a methodology for improving material assessment reporting across independent research efforts by providing the tools to evaluate contributions from sample preparation, handling, and method performance for a broad range of in-house material controls and advance material assessment across applied .

## Supplementary Material



## Data Availability

All raw vibrational spectra will be available upon reasonable request from the authors.

## Footnotes

1. Certain commercial products or equipment are described in this paper in order to specify adequately the experimental procedure. In no case does such identification imply recommendation or endorsement by the National Institute of Standards and Technology, nor does it imply that it is necessarily the best available for the purpose.

## Notes

Additional experimental details, tables of experimental parameters used in each measurement, statistical analysis of crystallinity between samples, and Figures S1–S19 (PDF)
