# Supplementary material for: Spectroscopically Evaluating Composition and Crystallinity in Pristine and UV-Exposed PET Films Using Polarization-Dependent Optical Photothermal Infrared Microscopy
Source: Anal Chem. 2026 Sep 25;98(40):29162–73. doi: 10.1021/acs.analchem.6c01918 (PMC13670890; doi:10.1021/acs.analchem.6c01918)
Supplement: Supplementary file 1 [file ac6c01918_si_001.pdf]

Spectroscopically evaluating composition and crystallinity in pristine and UV-exposed PET films using polarization dependent Optical Photothermal Infrared Microscopy

Danuta Liberda-Matyja,[1,2] Hsiu-Chin Huang,[3,4] Tae Joon Cho,[1] Li-Piin Sung,[3] and John Pettibone[1]**

[1]Materials Measurement Science Division, National Institute of Standards and Technology, Gaithersburg, MD, 20899, USA

[2]Department of Chemistry and Biochemistry, University of Maryland, College Park, MD, 20742, USA

[3]Materials and Structural Systems Division, Engineering Laboratory, National Institute of Standards and Technology, Gaithersburg, MD, 20899, USA

[4]Department of Chemistry, Krieger School of Arts and Sciences, Johns Hopkins University, Baltimore, MD, 21218, USA

Corresponding authors*: Email: danuta.liberdamatyja@nist.gov, john.pettibone@nist.gov

Table of Contents:

Additional details for data processing and analysis

Table S1. Experimental parameters and acquisition settings for all OPTIR measurements

Table S2. Descriptive statistics (means and standard deviations) and corresponding pairwise significance levels (p-values) for PET samples of varying crystallinity.

Figures S1-S19

Additional details for data processing and analysis

Sample cross-sectioning induced molecular orientation analysis

To rigorously test and validate *k* factor intensity mapping presented in Figure 2 of the main text, the entire control OPTIR dataset was independently analyzed using Multivariate Curve Resolution - Alternating Least Squares (MCR-ALS) as an unsupervised chemometric benchmark (Figure S7 and S8). For the intermediate drawn regimes (Stretched 1 and 2), the calculated *T* conformer fractions were nearly identical between the two methods (varying by less than 2%, Fig. S8). For the highly amorphous sample baseline, the univariate approach yields a *T* fraction equal to 13%, which was closer to the reported literature framework for relaxed amorphous chains than the MCR-ALS estimation (21 %). Furthermore, both models result in high goodness of fit for the complex, broadened multi-modal distribution profiles observed in the microtome and blade cross-sectioned specimens, with the microtome *T* fraction centering consistently around 26-28 % in both plots (Fig. S8 A and C).

PCA analysis description of PET films

In the loading plots (Fig. S5), the two components separate into a chemical and an orientational axis. PC2 captures crystallinity: it is driven by the crystalline marker bands—the narrow, downshifted carbonyl at 1717 cm^{-1}, the trans CH_2 wagging mode at 1345 cm^{-1}, and the ring-ester in-plane mode at 1131 cm^{-1}—and separated the crystalline samples (annealed, biaxial) from the rest.[1] PC1 captures molecular orientation: it is driven by the dichroic, carbonyl band at 1729 cm^{-1}, CH_2 wagging mode at 1345 cm^{-1} and the in-plane ring/ester modes near 1101 cm^{-1}—whose loadings varied with polarization angle, consistent with the perpendicular and parallel dichroism reported by Cole et al.[1]

Critically, the loading shapes are conserved across all five polarization angles, while only their amplitudes changed. This result demonstrated that the same chemical signatures contribute to the variance at every angle, and that the angle-dependent modulation reflects molecular orientation (dichroism) rather than a change in chemical composition.

Single Polarization versus five polarization data using 2T2D Analysis

To demonstrate the applicability and representative nature of the 0p approach, a comparative 2T2D analysis was performed using the average of five polarizations (5p) and the single polarization, 0°, dataset measured on the 35d UV-exposed PET sample. Spectral data were collected using the following parameters: 4% nominal IR power, 40x objective, 20x detector gain, across a cross-sectional area of 16 µm width by 34 µm height. Mapping was done with an asymmetrical step interval of 0.5 µm horizontally and 1 µm vertically down the depth axis (1845–919 cm^{-1} range) using the same preprocessing that was applied to data in Fig. 5.

The results across all panels (Figs. S15 and S16 A–E) demonstrated that the 0p approach was sufficient to capture the photo-oxidation gradient and chemical weathering features in the 5p data while reducing instrument acquisition time by 80%. Specifically, both datasets exhibited the

identical three-layer structure with K-means clustering (Panel C), which supported successful identification of the highly degraded surface region (Cluster 1, 0–3 µm), the sub-surface (Cluster 2, 3–7 µm), and the least modified bulk polymer (Cluster 3, >7 µm). However, the continuous depth profiles of the (1343, 1375) cm^{-1} band pair (Panel D, pink curve) were affected by the microtome-induced orientation. When comparing the 0p data against the 5p average, both a distinct spatial shift and a larger standard deviation became apparent. In the 5p dataset, the pink curve rises more gradually to a broad maximum at approximately 15 µm, whereas the 0p curve grows faster, reaching its peak intensity near 12 µm before decreasing down the depth axis.

There are other possible sources for the observed compositional variations. First, the 5p average mixes the strong horizontal mesomorphic alignment from the microtome into the profile, which may blur the boundary between weather-induced crystallization and the pristine core, pushing the perceived plateau deeper. By operating perpendicular to the cutting axis, the 0p might avoid this horizontal artifact, which would result in a depth-limit of the UV-degradation zone earlier. Conversely, if the UV-induced crystallinity in the upper layers (0–7 µm) develops transition dipole moments oriented perpendicular to the 0° probe axis, this shift could instead reflect a loss of intensity resulting from molecular orientation rather than a true change in the physical chemical front. Additionally, the larger error bands in the 0p profile directly reflected the high sensitivity of a single polarization to local variations in polymer chain orientation. Unlike the 5p average, which should dampen structural fluctuations by combining multiple angles, the single-angle mode lacks this smoothing effect, leaving both raw pixel noise and alignment changes observed along the depth profile.

Table S1. Experimental parameters and acquisition settings for all OPTIR measurements.

Parameter Category	Specific Parameter	Experimental Details
**System & Optics**	Instrument	Commercial OPTIR mIRage-LS system (Photothermal Spectroscopy Corp.)
	Measurement Mode	Co-propagating mode
	Objective	40x Cassegrain objective (0.78 NA)
**Laser Settings**	Pump Laser (IR)	MIRcat polarized quantum cascade laser (QCL)
	Pump Pulse Rate & Power	100 kHz pulse rate, 8% power
	Probe Laser (Visible)	532 nm laser
	Probe Power & Gain	2.8% power, 20x standard detector gain
**Polarization**	Control Component	Wire grid polarizer
	Reference Angles	0° (parallel to intrinsic QCL)<br>90° (sample principal symmetry axis alignment)
	Measured Polarizations	Control/Cross-sectioned am-PET: 5 angles (0°, 45°, 60°, 120°, 135°)<br>Weathered Film: 0° only
**Spectral Data** **(Control films and Cross-sectioned am-PET)** Figure 2 and 3	Samples measured	Control films one sample per each category: amorphous, stretched1, stretched2, annealed and biaxial<br>Cross-sectioned films: two blade cut samples (blade1, blade2), one microtome cross sectioned sample
	Spectral Range	1807 – 957 cm^{-1}
	Spectral Resolution	6.6 cm^{-1}
	Scan Integration	4 scans per sample, 16 scans per background
	Focusing Method	OPTIR signal-based autofocusing
Sample & Sampling Size	Number of Spectra	100 spectra (points measured) per sample for control films. 80 spectra (equal number for each class) were used for analysis following outliers removal.<br>100 spectra per sample for cross-sectioned a-PET (100 points measured in 10x10 μm imaging area). 80 spectra (equal number for each class) were used for analysis following outliers removal.
	Location Points / Spots	100 random points on the surface (controls) and 100 points within the imaging area using a 1 μm x/y step size (cross-sections). Reproducibility was evaluated from n > 6 spots.
**Imaging Mode** **(Cross-sectioned am-PET)** Figure 4	Samples measured	Cross-sectioned films: two blade cut samples (blade1, blade2), one microtome cross sectioned sample
	Step Size & Scan Rate	10x10 μm, 200 nm step size, 80 pt/s scan rate (51x51 pixels)
	Chemical Contrast Points	1343 cm^{-1}, 1373 cm^{-1}, 1413 cm^{-1}
	Baseline Correction Points	1331 cm^{-1}, 1363 cm^{-1}, 1391 cm^{-1}, 1431 cm^{-1}
	Focusing Method	Minimizing probe beam spot size on sample surface
**Hyperspectral Mode** **(Weathered Film)** Figure 5	Samples measured	One sample UV exposed for 35 days (1 μm x 1 μm step (39 μm x 59 μm))
	Spectral Range and resolution, Scan Integration	Same as controls
	Focusing Method	Minimizing probe beam spot size on sample surface
	Number of spectra	2127 spectra after outliers removal

Table S2. Descriptive statistics (means and standard deviations) and corresponding pairwise significance levels (p-values) for PET samples of varying crystallinity.

Group_A	Group_B	Mean_A	Std_A	Mean_B	Std_B	p_value
**am-PET**	stretched1	0.683	0.033	1.780	0.096	< 0.001
**am-PET**	stretched2	0.683	0.033	1.992	0.136	< 0.001
**am-PET**	annealed	0.683	0.033	4.561	0.528	< 0.001
**am-PET**	biaxial	0.683	0.033	9.441	0.651	< 0.001
**stretched1**	stretched2	1.780	0.096	1.992	0.136	< 0.001
**stretched1**	annealed	1.780	0.096	4.561	0.528	< 0.001
**stretched1**	biaxial	1.780	0.096	9.441	0.651	< 0.001
**stretched2**	annealed	1.992	0.136	4.561	0.528	< 0.001
**stretched2**	biaxial	1.992	0.136	9.441	0.651	< 0.001
**annealed**	biaxial	4.561	0.528	9.441	0.651	< 0.001

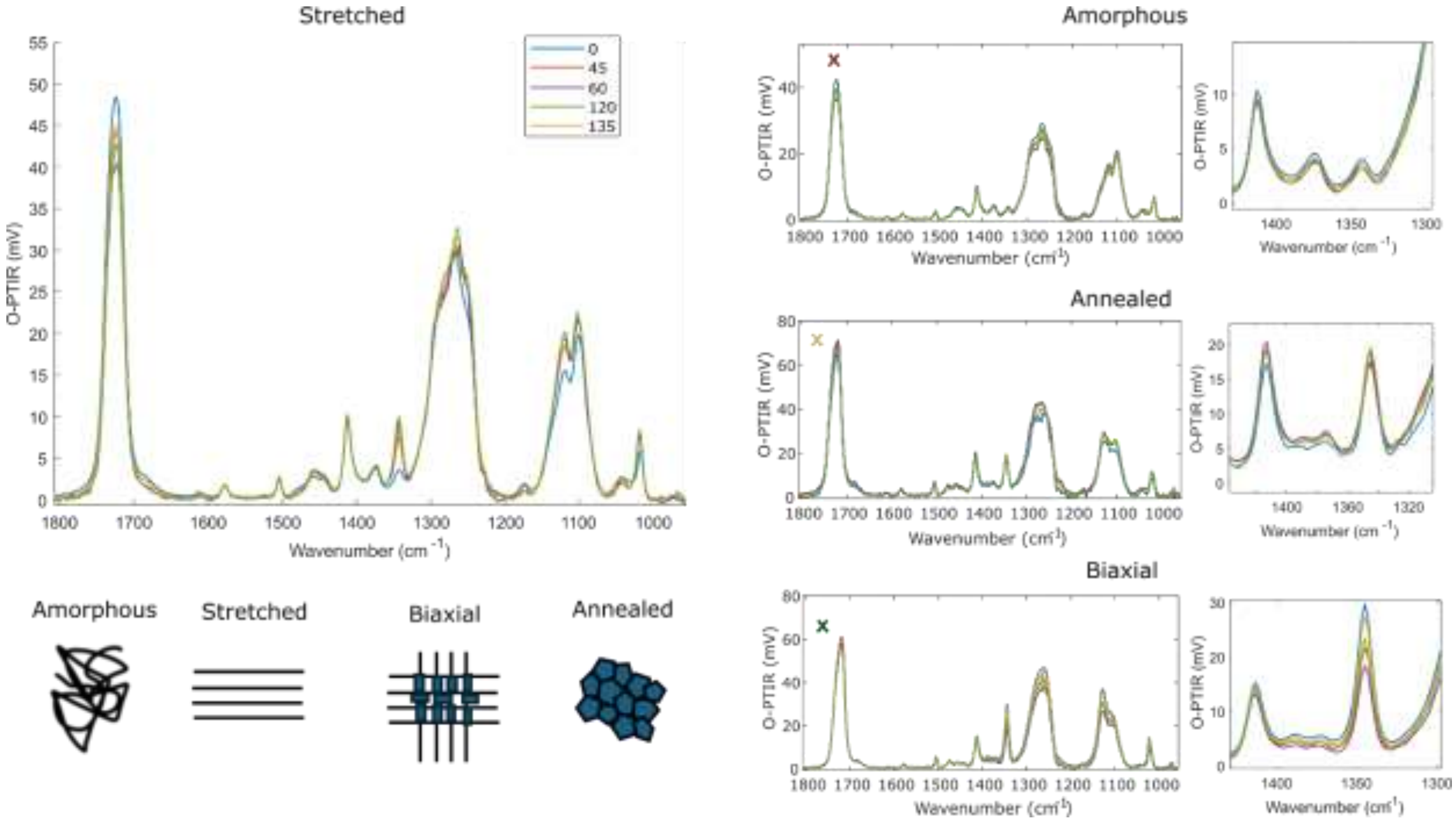

Figure S1. Raw polarized spectra measured from the sample location identified in Fig. S4 at each polarization angle with magnified view of the spectral region for the *G* and *T* glycol conformers from (1300-1500) cm^{-1}.

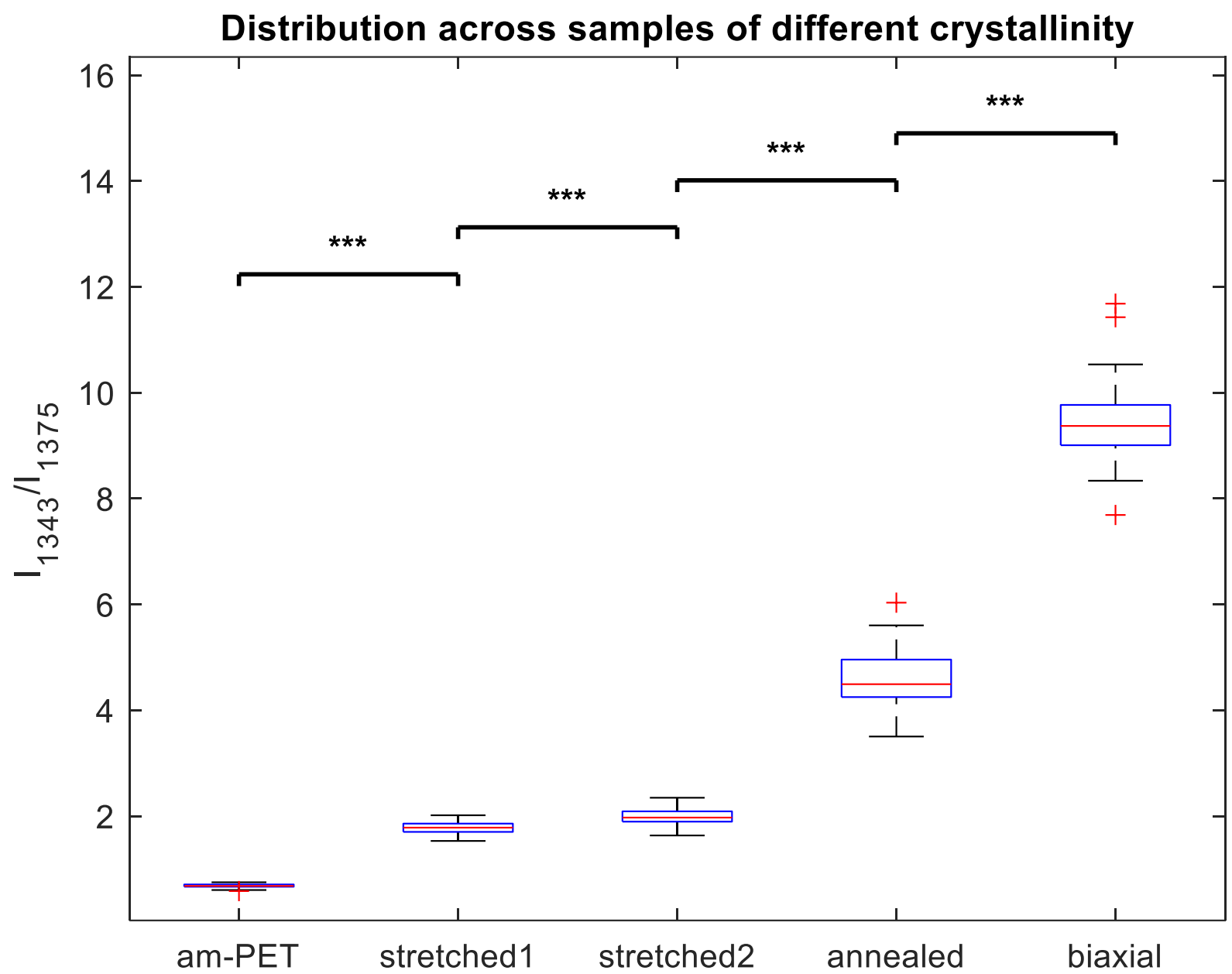

Figure S2. A statistical comparison of the distribution of I_{1343}/I_{1375} across five distinct PET samples. Box plots indicate the median, quartiles, and range (n = 80 spectra per group), with overlaid brackets indicating significant pairwise differences between each PET sample (*p < 0.05, **p < 0.01, ***p < 0.001).

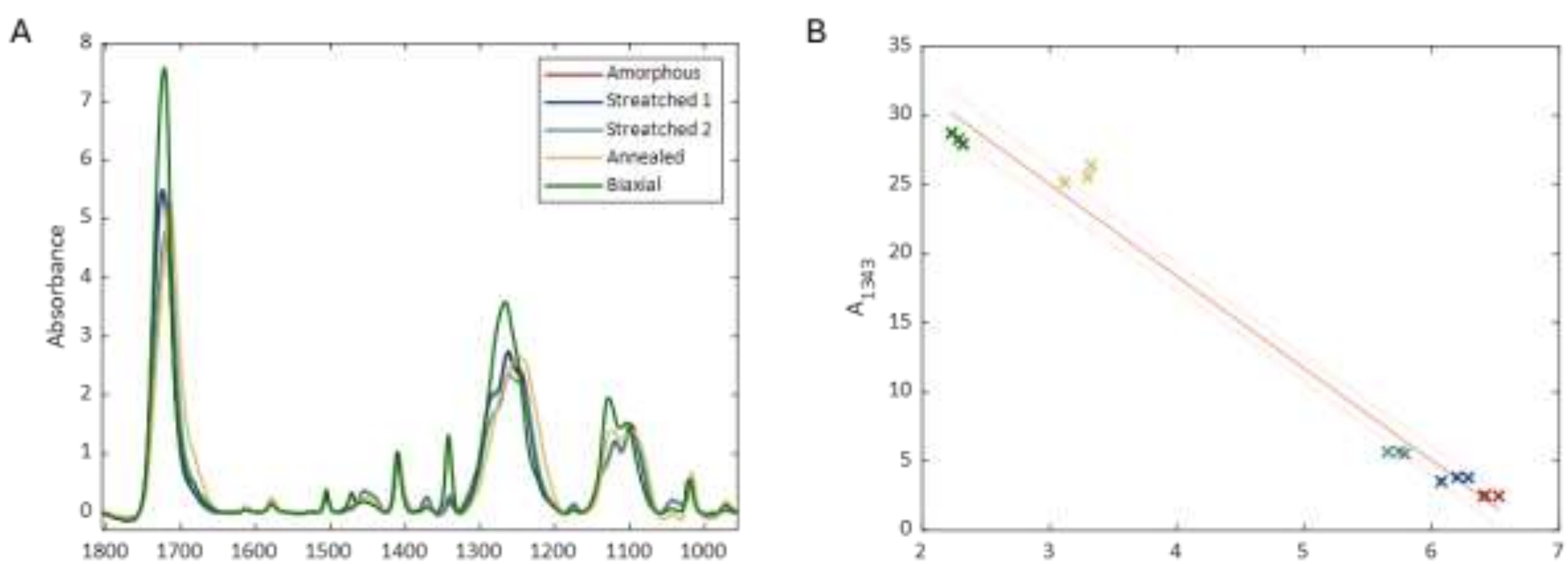

Figure S3. A) Averaged ATR-IR spectra and B) integrated-absorbance fit. The "×" points in panel B) are color-coded to the sample legend in panel A), the dashed curves around the regression line represent the 95% confidence bounds for the fit. Regression statistics: $R^2 = 0.982$, RMSE = 1.61, F = 705, $p = 1.04 \times 10^{-12}$, $n = 15$.

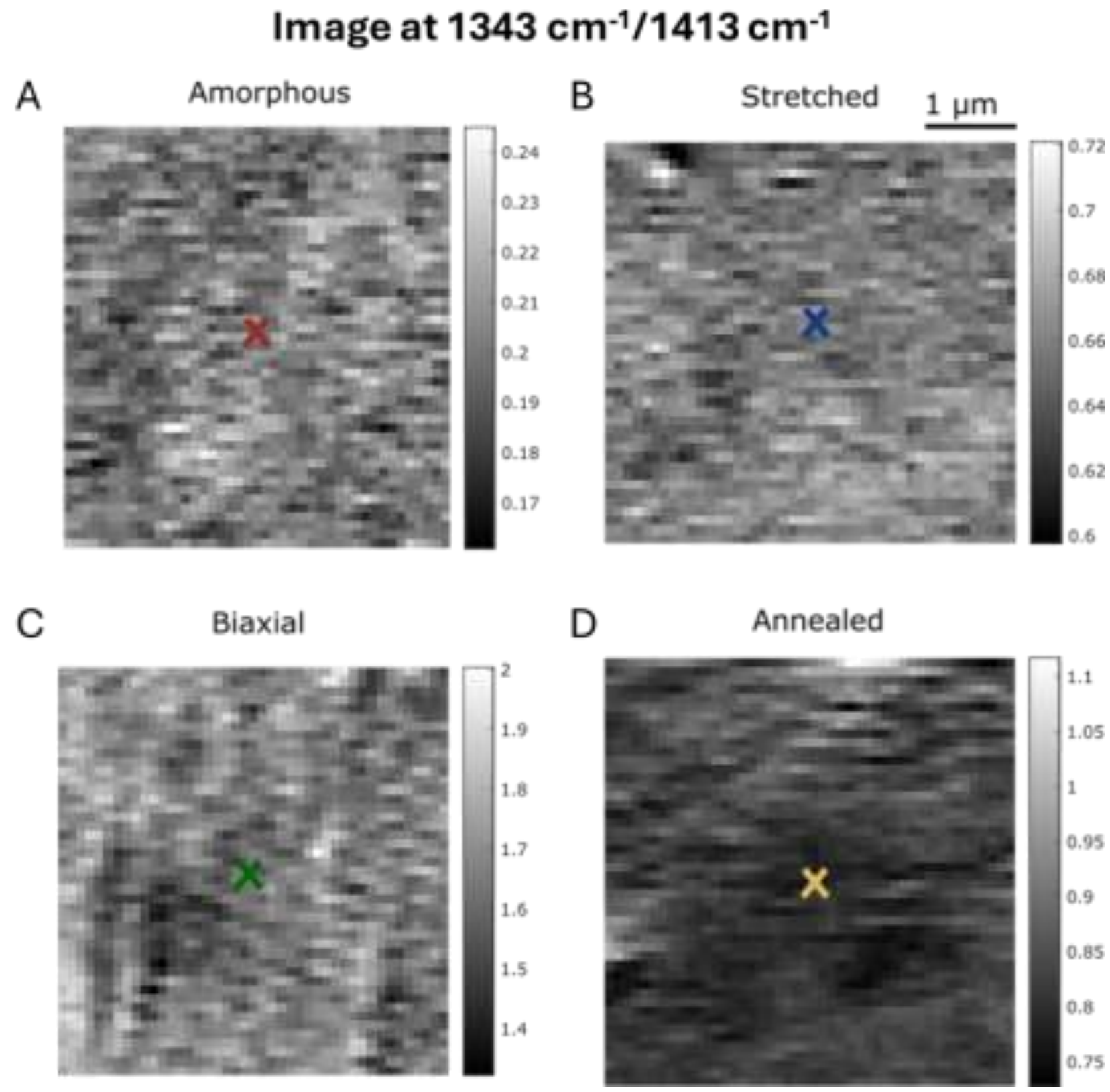

Figure S4. Baseline corrected and aligned 5p averaged images normalized to 1413 cm^{-1} for control samples: A) amorphous, B) stretched, C) biaxial and D) annealed. The “X” represents the position of 5p spectra measurement location for each image presented in Figure 1.

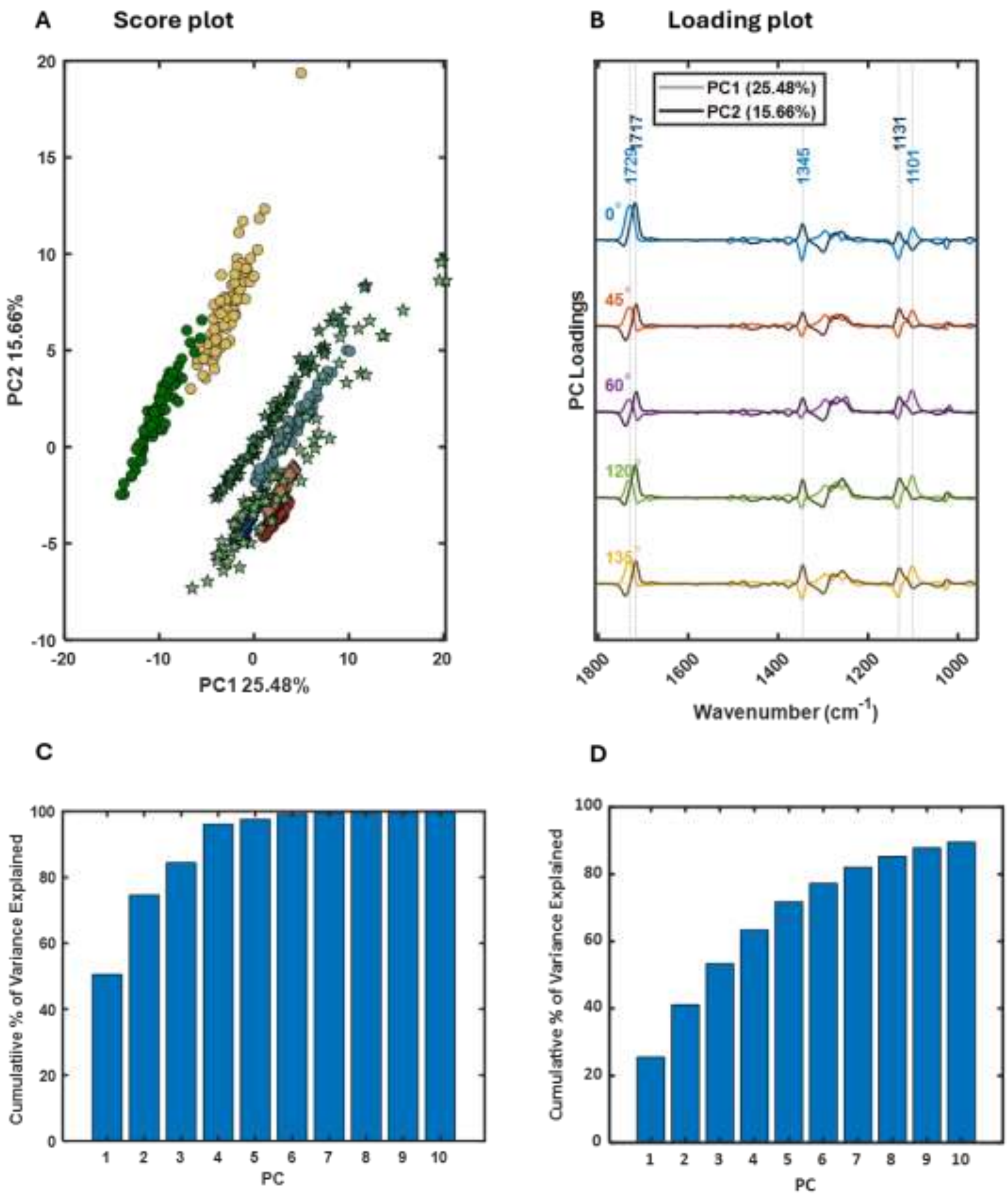

Figure S5. Principal component analysis (PCA) of infrared spectra detailing structural modifications in PET across multiple polarization angles. A) PCA scores plot, B) Stacked loading plots for PC1 (brighter lines) and PC2 (darker lines) tracked across five distinct infrared polarization angles (0°, 45°, 60°, 120°, and 135°) over the 957–1807 cm^{-1} fingerprint wavenumber range. Vertical dotted grid lines highlight the primary vibrational features driving structural variance across all groups: crystalline ester carbonyl stretching (1717 cm^{-1}), oriented/amorphous carbonyl stretching (1729 cm^{-1}), the glycol trans conformer CH_2 wagging mode (1345 cm^{-1}), crystalline ring-ester in-plane mode (1131 cm^{-1}), and an in-plane ring mode(1101 cm^{-1}). Annotations for tightly adjacent critical frequencies are vertically staggered above the top 0° polarization. C) Cumulative % of variance for average spectra based PCA (Figure 3), D) Cumulative % of variance for 5p spectra based PCA. Each point on the score plot represents one spectrum. A total of 80 spectra per sample (amorphous, stretched 1, stretched 2, annealed, biaxial, microtome, blade 1, and blade 2) are shown, following the removal of outliers.

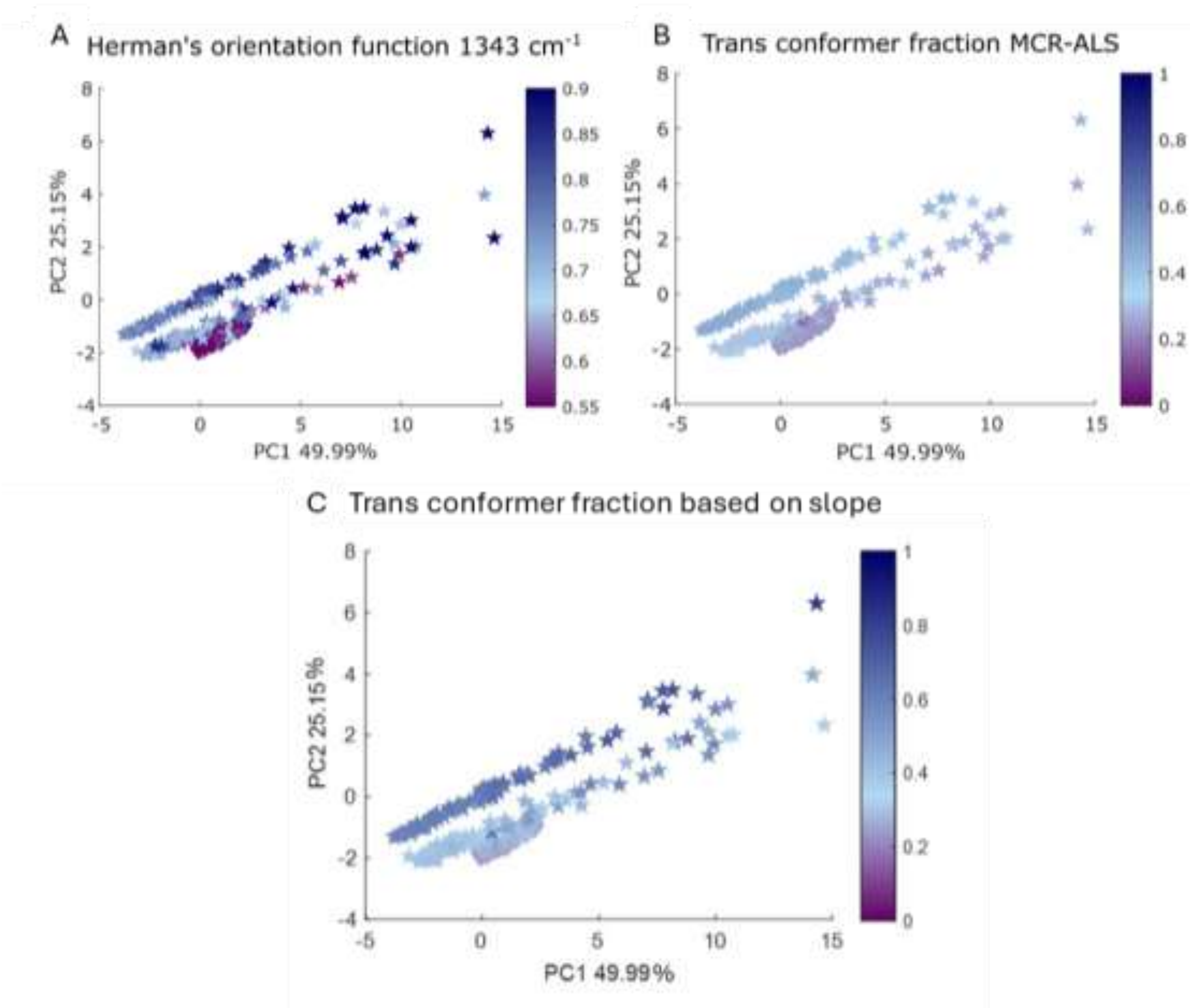

Figure S6. PCA on PET films and cross-sectioned samples with corresponding scores projection; outputs were color-coded based on: A) Hermans orientation function at 1343cm^{-1}, B) trans conformer fraction results from MCR-ALS, and C) trans conformer fraction based on two bands intensity method.

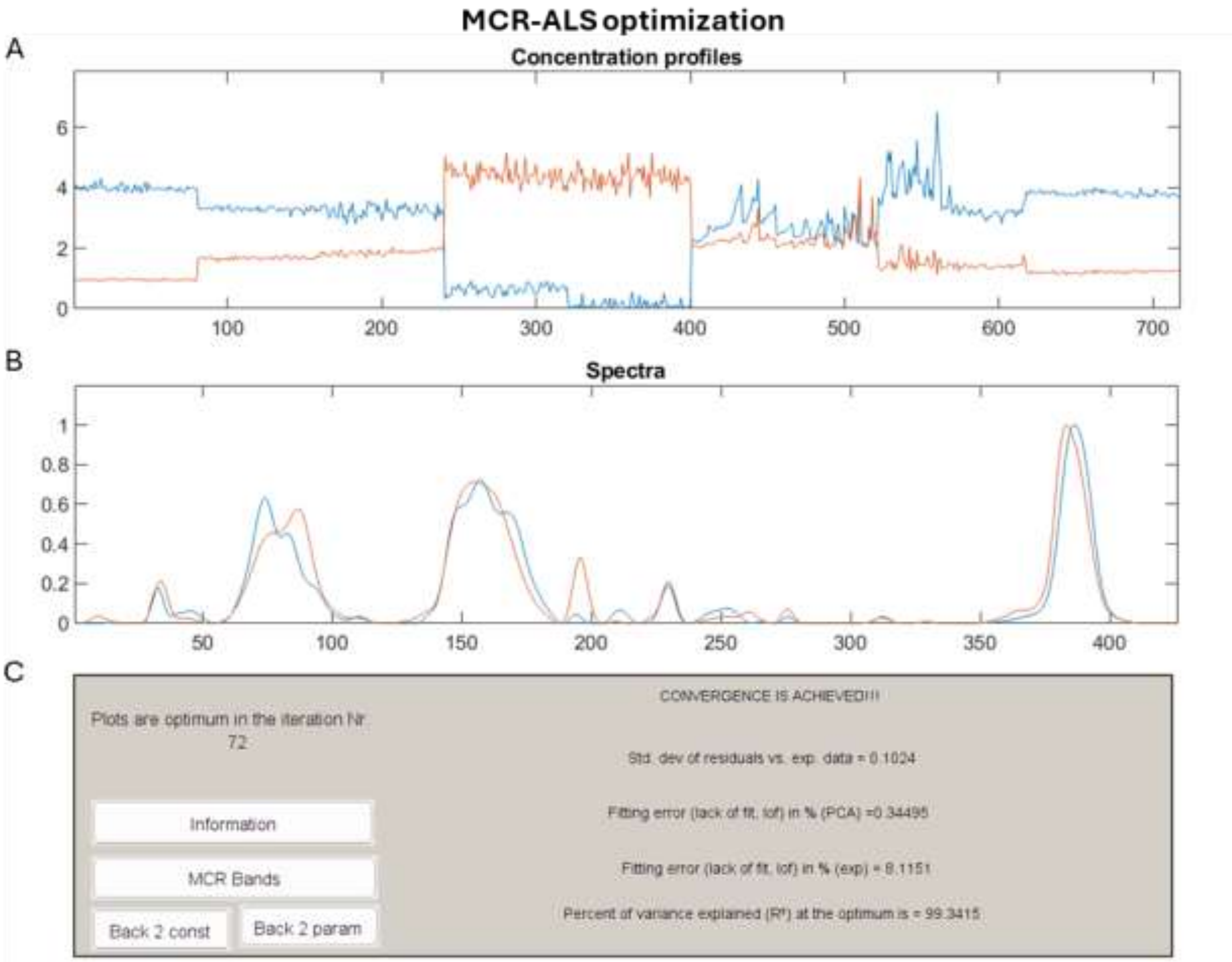

Figure S7. MCR-ALS optimization results for trans (orange) and gauche (blue) glycol conformers that were represented by: A) concentration profiles, B) spectra and C) performance evaluation metrics (mcr_toolbox2 plot). Input samples were as follows: 1:80 amorphous, 81:160 stretched 1, 161:240 stretched 2, 241:320 annealed, 321:400 biaxial, 401:522 blade 2, 523:616 blade 1, and 617:717 microtome.

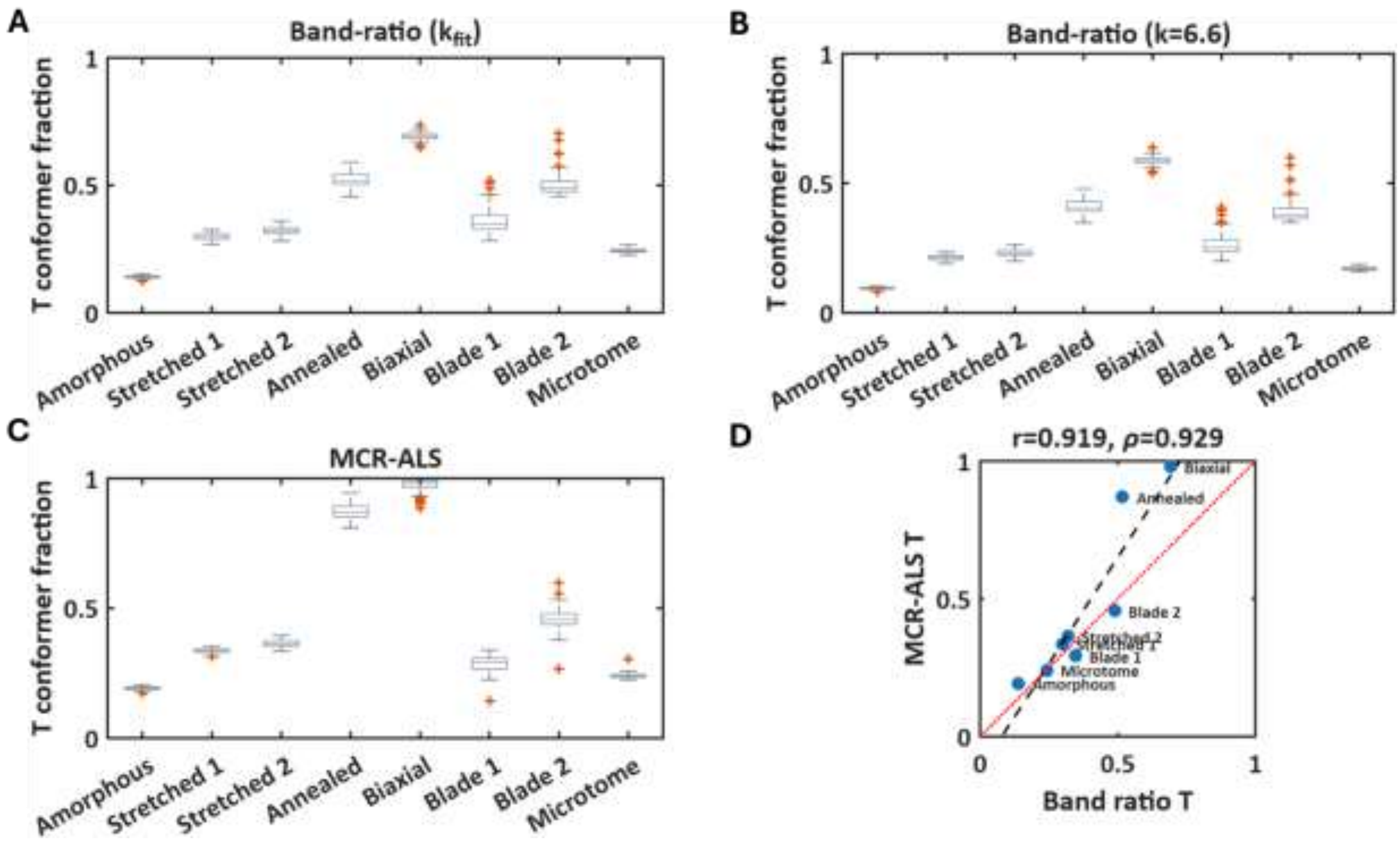

Figure S8. T conformer fraction comparison between band ratio and MCR-ALS based approaches: A) band ratio calculated with k=4.2, B) band ratio calculated with literature value k=6.6, C) MCR-ALS results, and D) parity plot comparing median T conformer fraction (k=4.2) estimates from the band ratio method and MCR-ALS. The dashed pink line indicates the 1:1 reference, the dashed black line shows the linear regression fit. Pearson (r) and Spearman (ρ) correlation coefficients are given.

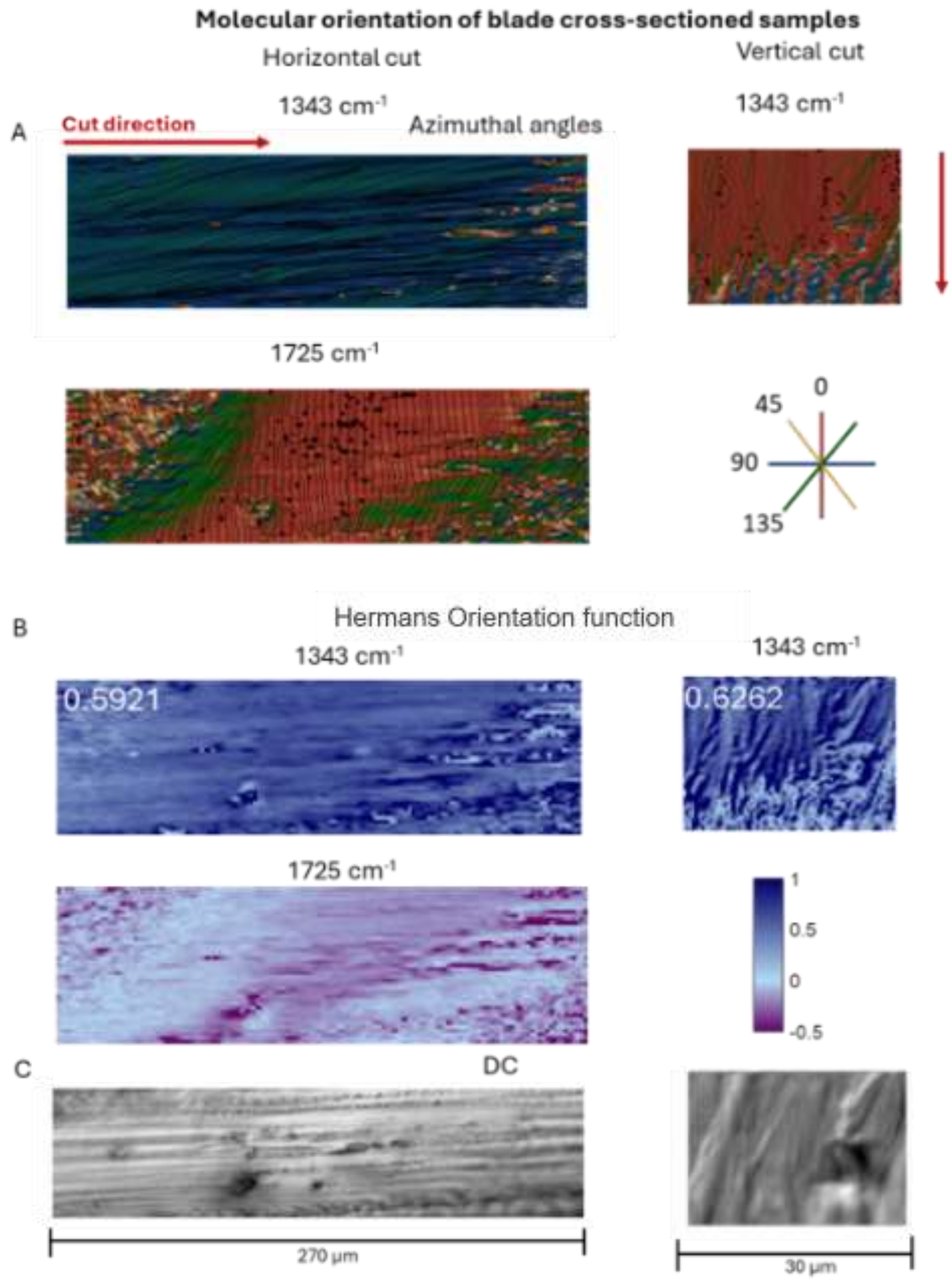

Figure S9. 5p analysis results for am-PET cross-sectioned by blade cut in horizontal and vertical direction: A) Azimuthal angle, B) Hermans orientation function with median value, and C) DC image (total scattering) of the surface. Measurement step 400 nm.

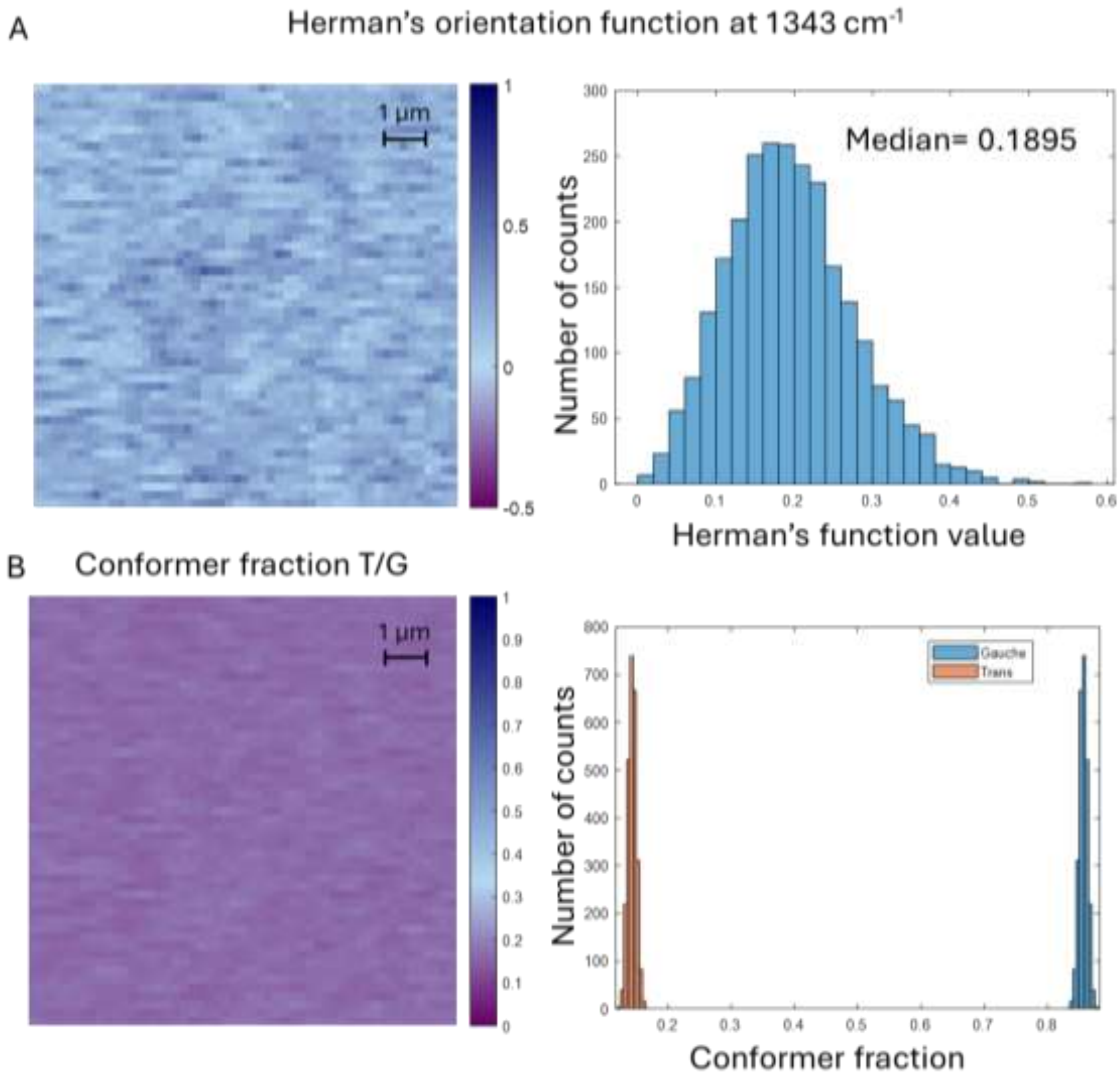

Figure S10. Hermans orientation function values for am-PET samples prepared by microtome and B) corresponding conformer fractions calculated at 1343 cm^{-1}.

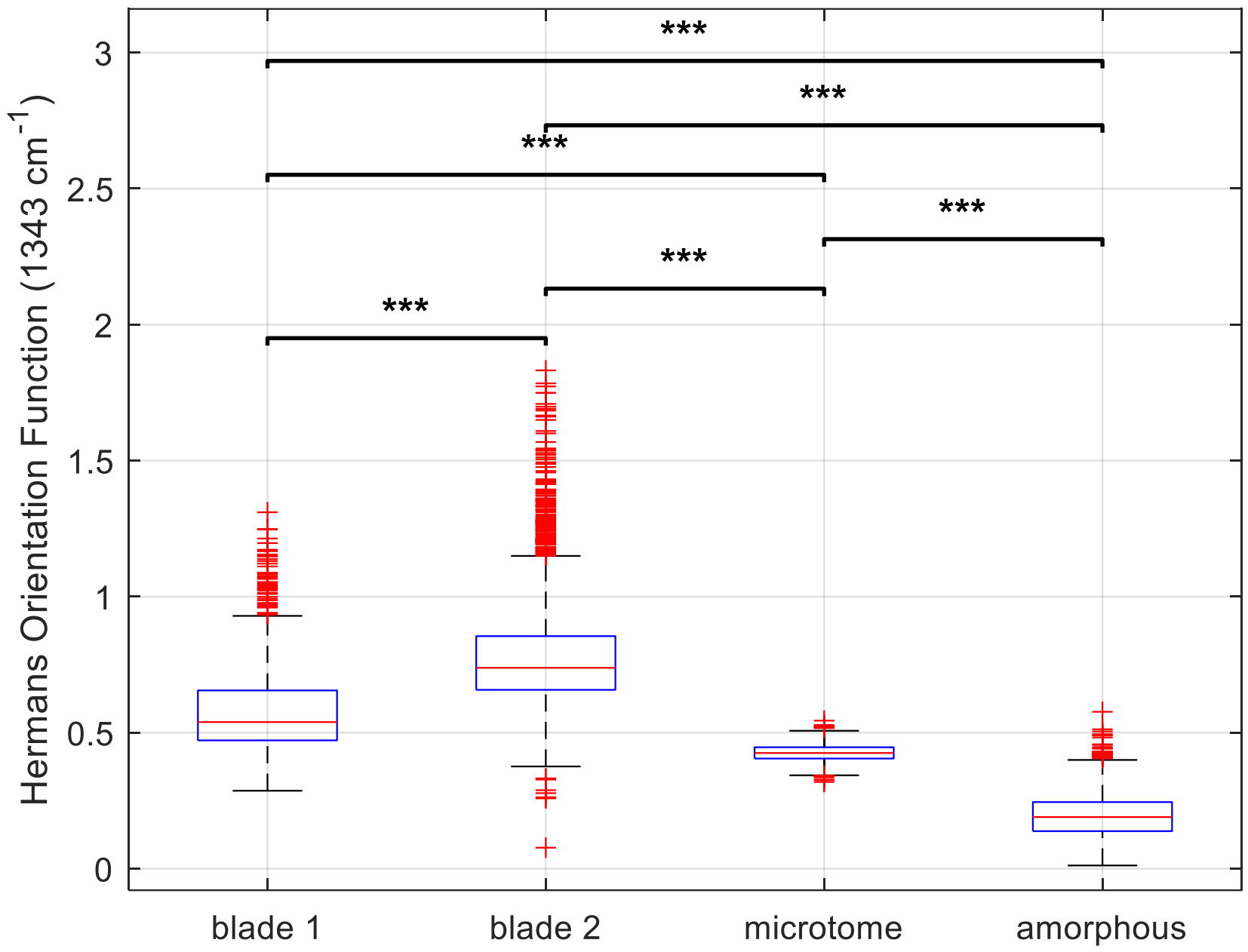

Figure S11. Comparison of the Hermans orientation function for PET samples across different surface preparation methods (blade 1, blade2, microtome, and amorphous). Statistical significance was determined using a two-sample Student's t-test (*p<0.05, *p < 0.05, **p < 0.01, *** p < 0.001).

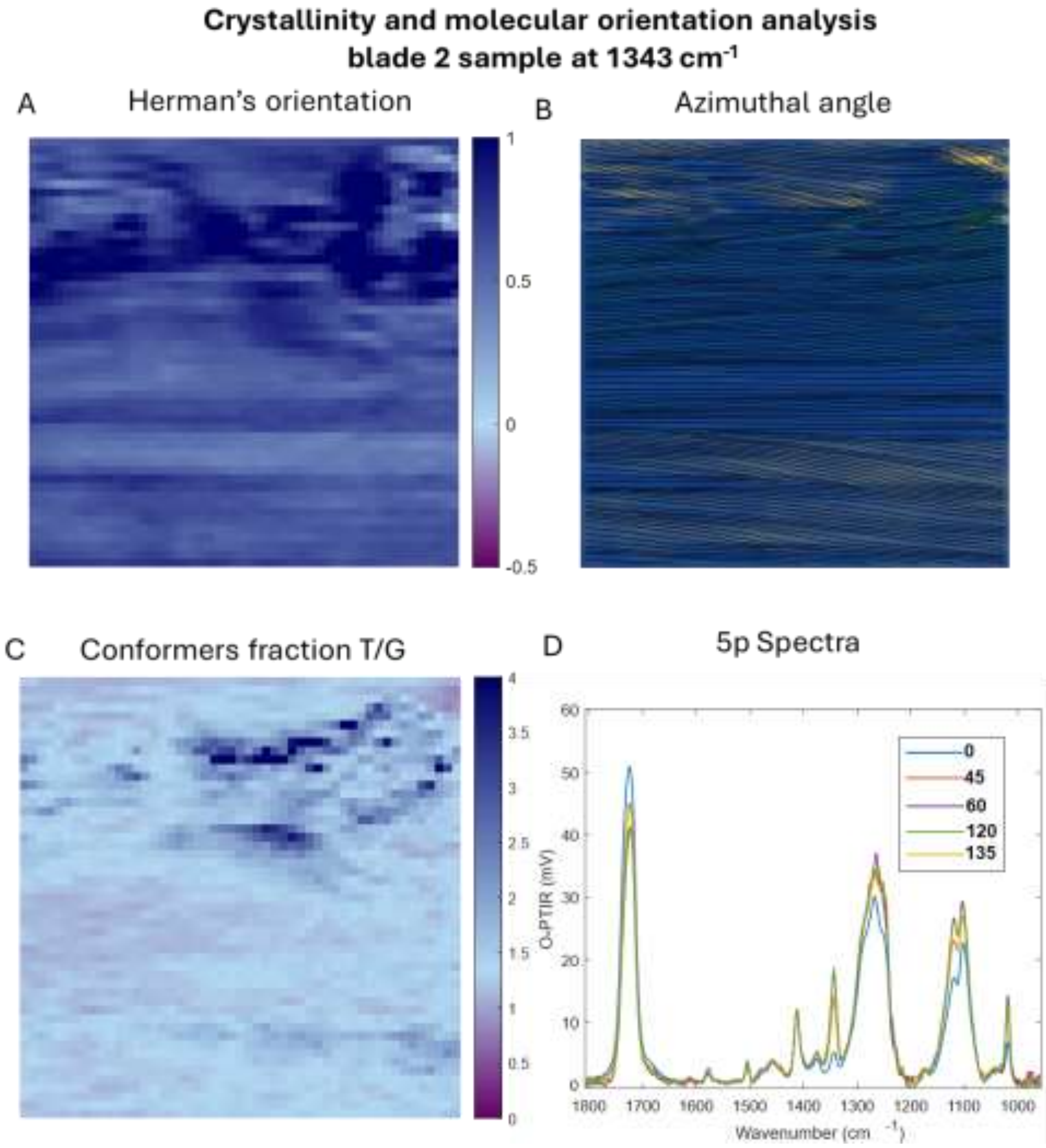

Figure S12. Crystallinity and molecular orientation analysis for 'blade 2' sample based on: A) Herman's orientation function, B) Azimuthal angles and C) conformer fractions ratio, along with D) 5p polarized spectra.

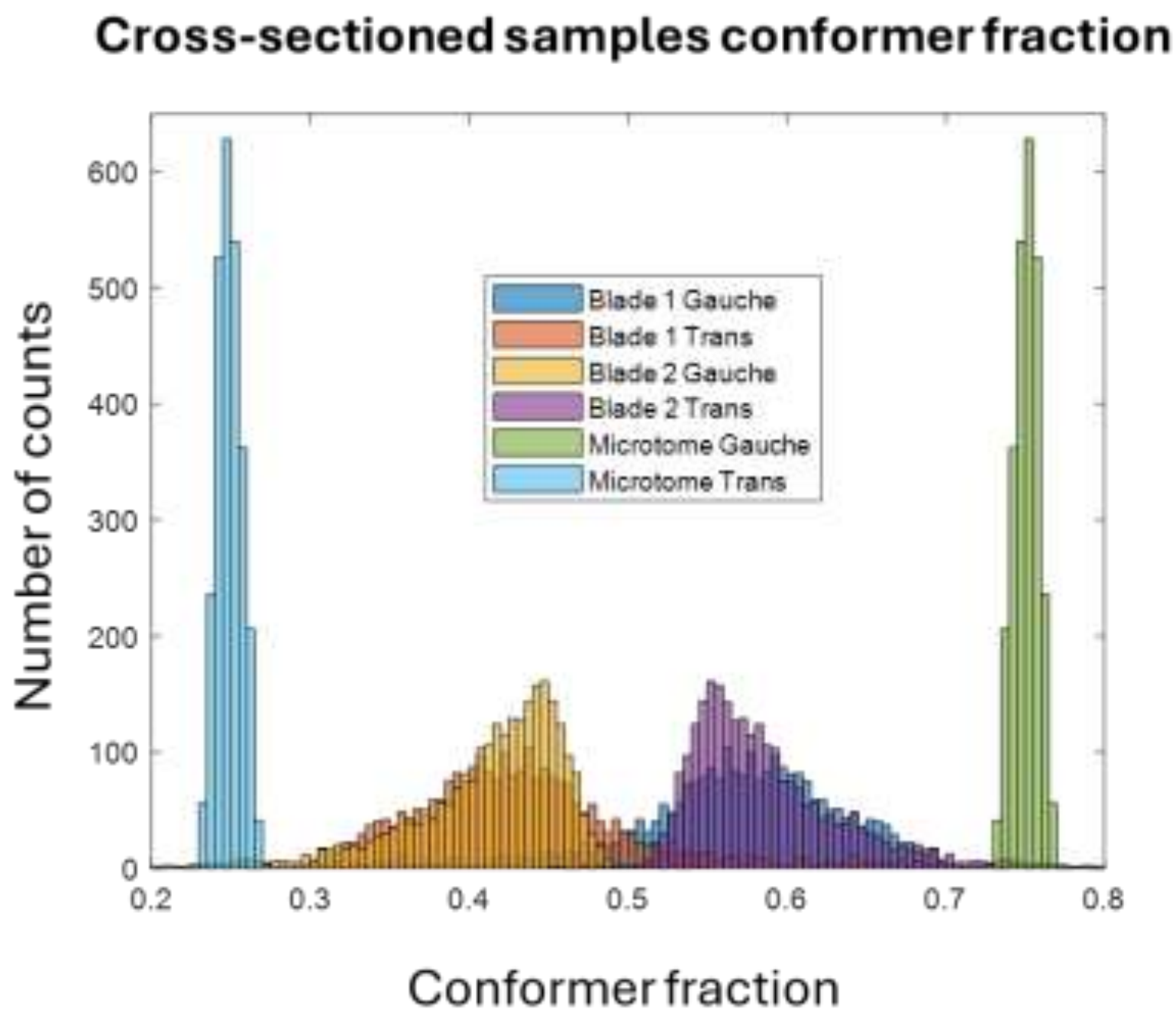

Figure S13. Histograms of calculated conformer fractions for imaged cross-sectioned samples including 'blade 2'.

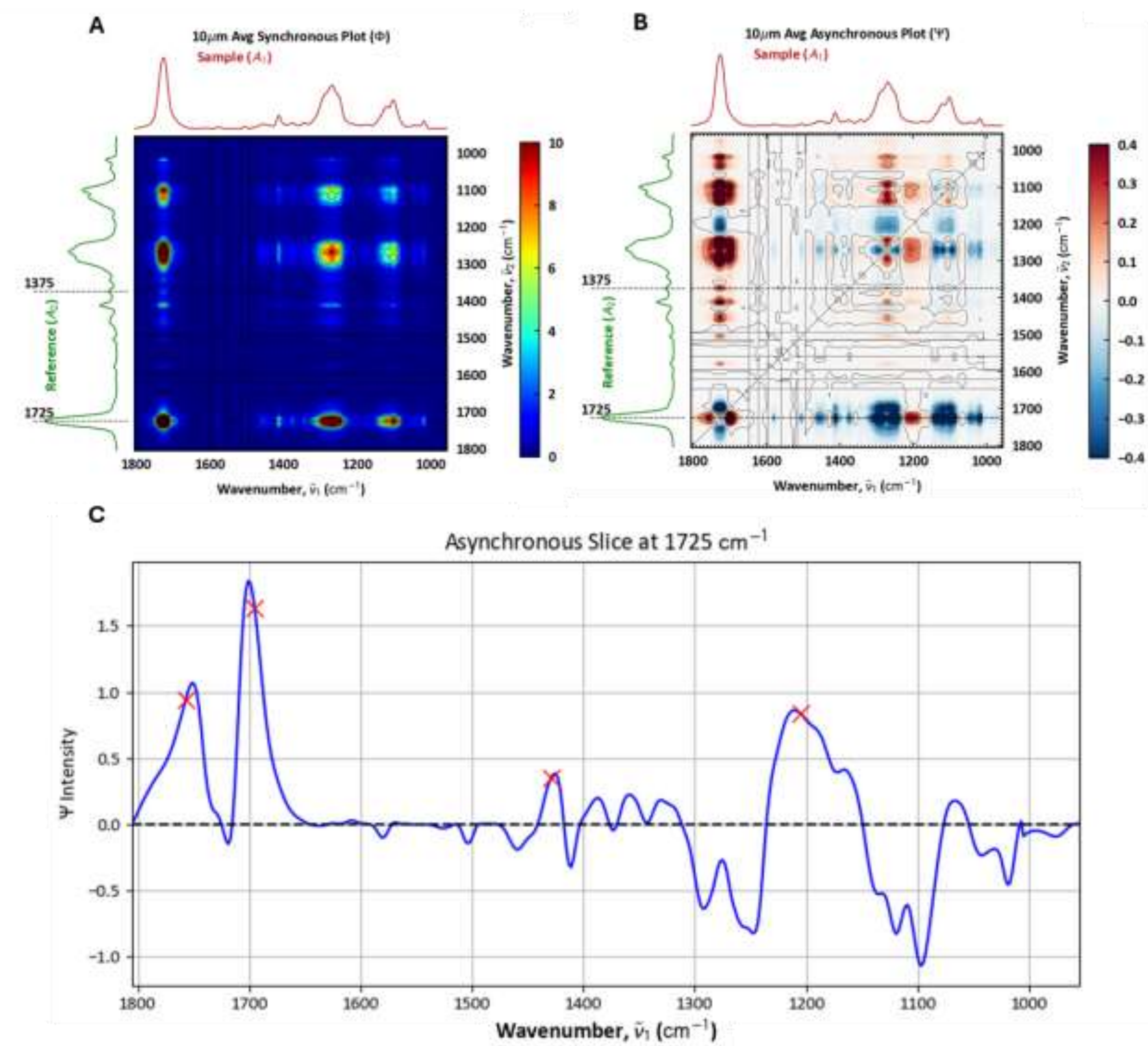

Figure S14. 2T2D correlation maps generated from the average spectrum for the 0–10 μm depth: A) synchronous, Φ, B) asynchronous, Ψ, and C) asynchronous intensity slice at 1725 cm^{-1}. Frequencies used for 2T2D analysis are marked with 'X'.

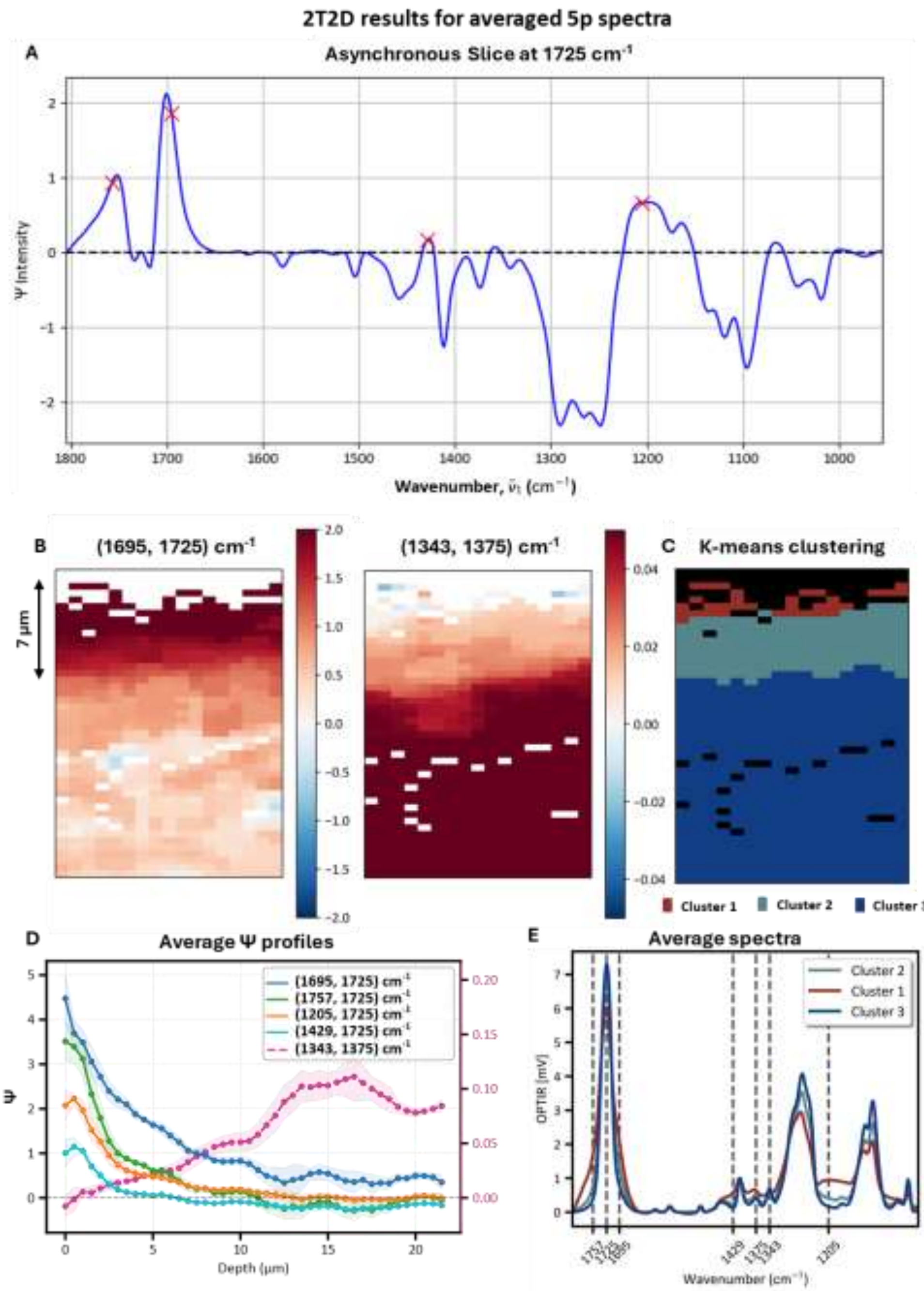

Figure S15. 2T2D correlation analysis for 5p averaged spectra (685 spectra after outliers removal): A) asynchronous intensity slice at 1725cm^{-1} generated from the average spectrum for the 0–10 µm depth, B) spatial 2T2D asynchronous (Ψ) maps, C) K-means clustering based on 2T2D results, D) averaged 1D asynchronous depth profiles plotted as a function of distance from the UV-exposed surface (0 µm), and E) cluster average spectra with wavenumbers of interest identified with dashed lines.

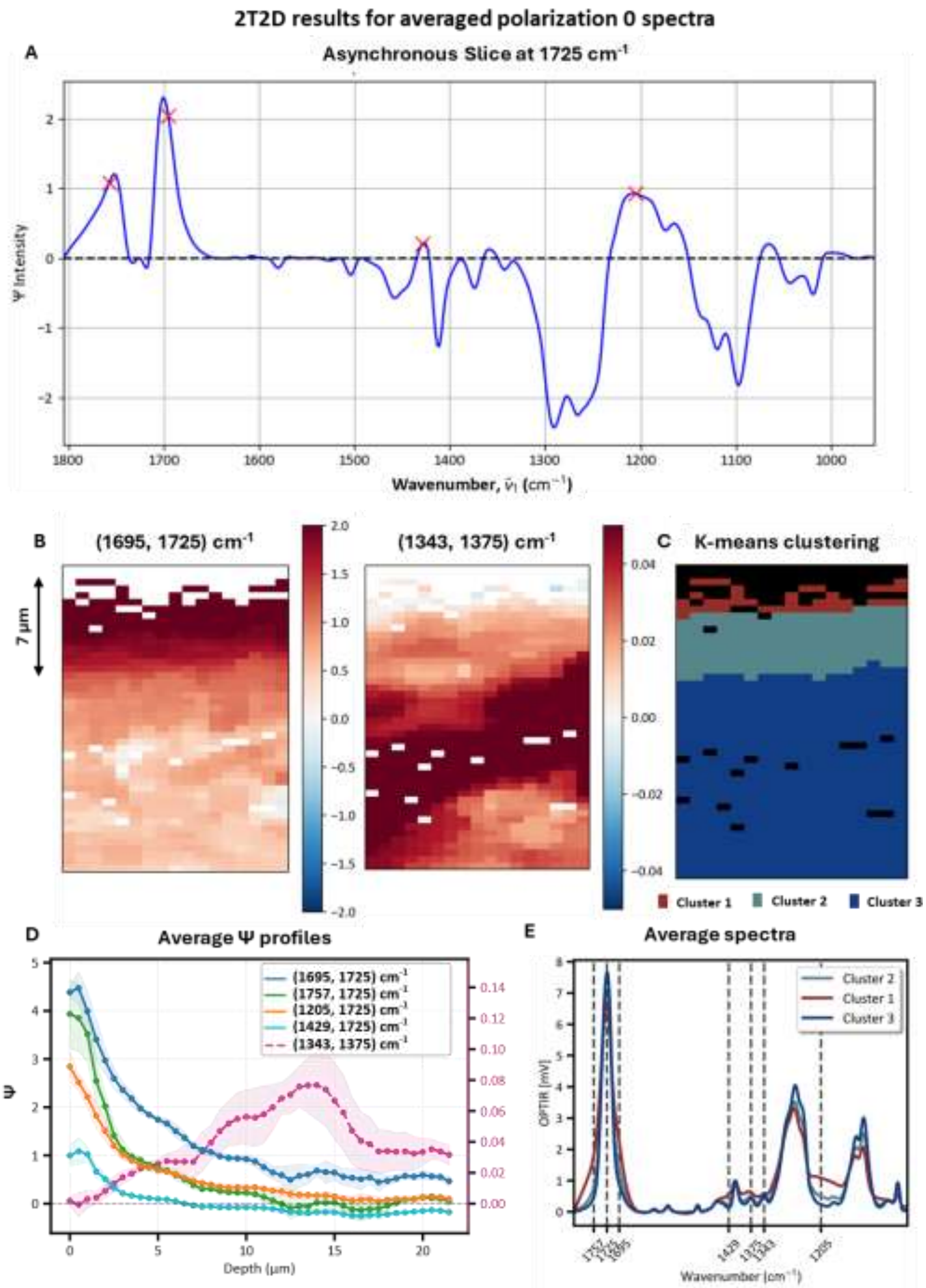

Figure S16. 2T2D correlation analysis for spectra measured with polarization 0° (689 spectra after outliers removal): A) asynchronous intensity slice at 1725cm^{-1} generated from the average spectrum for the 0–10 µm depth, B) spatial 2T2D asynchronous (Ψ) maps, C) K-means clustering based on 2T2D results, D) averaged 1D asynchronous depth profiles plotted as a function of distance from the UV-exposed surface (0 µm), and E) cluster average spectra with wavenumbers of interest marked with dashed lines.

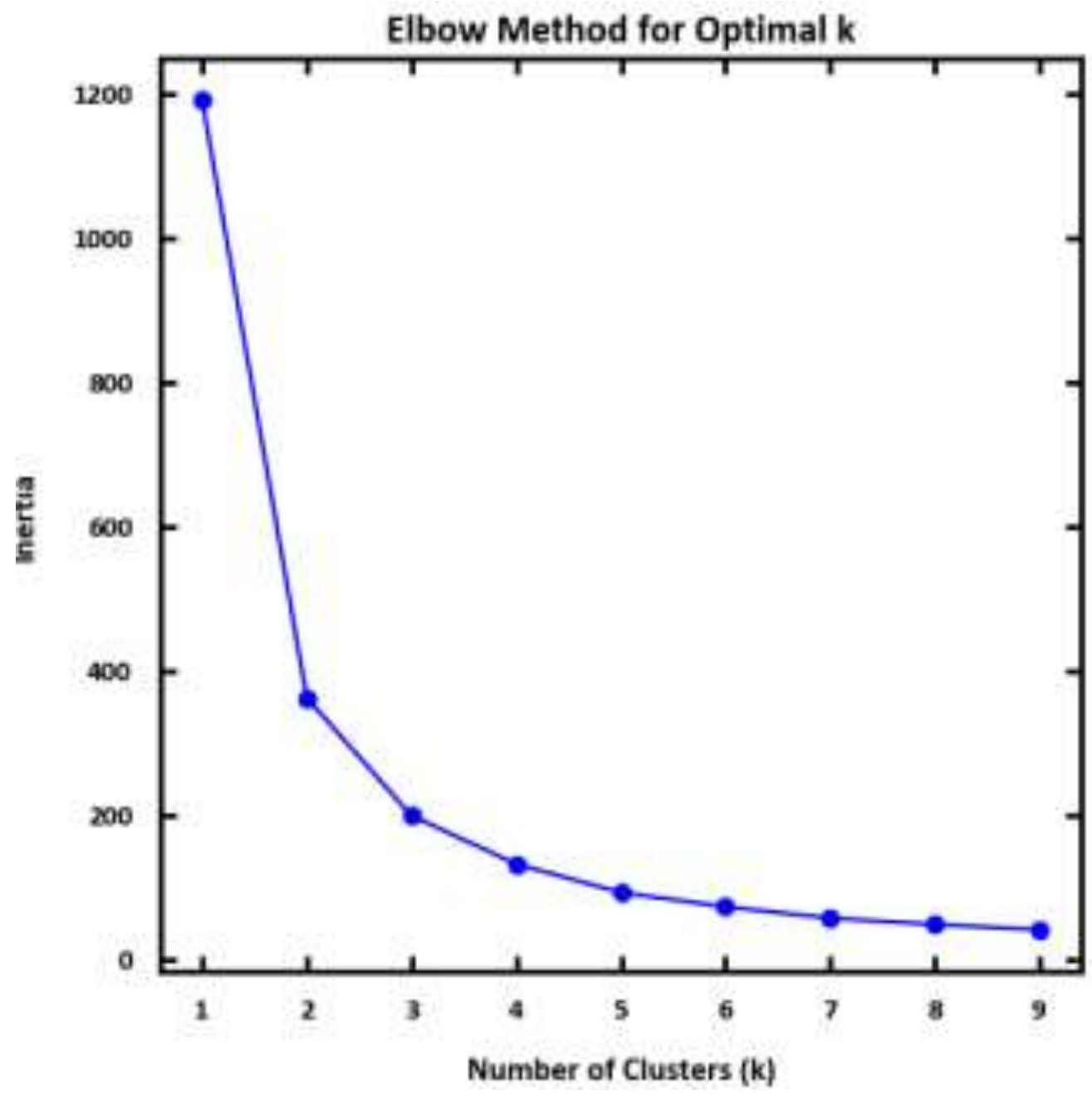

Figure S17. Evaluation of the optimal number of clusters (k) for K-means segmentation of the 2T2D asynchronous correlation data using the elbow method. The within-cluster sum of squares (inertia) was plotted as a function of k, indicating an optimal inflection point at $k = 3$.

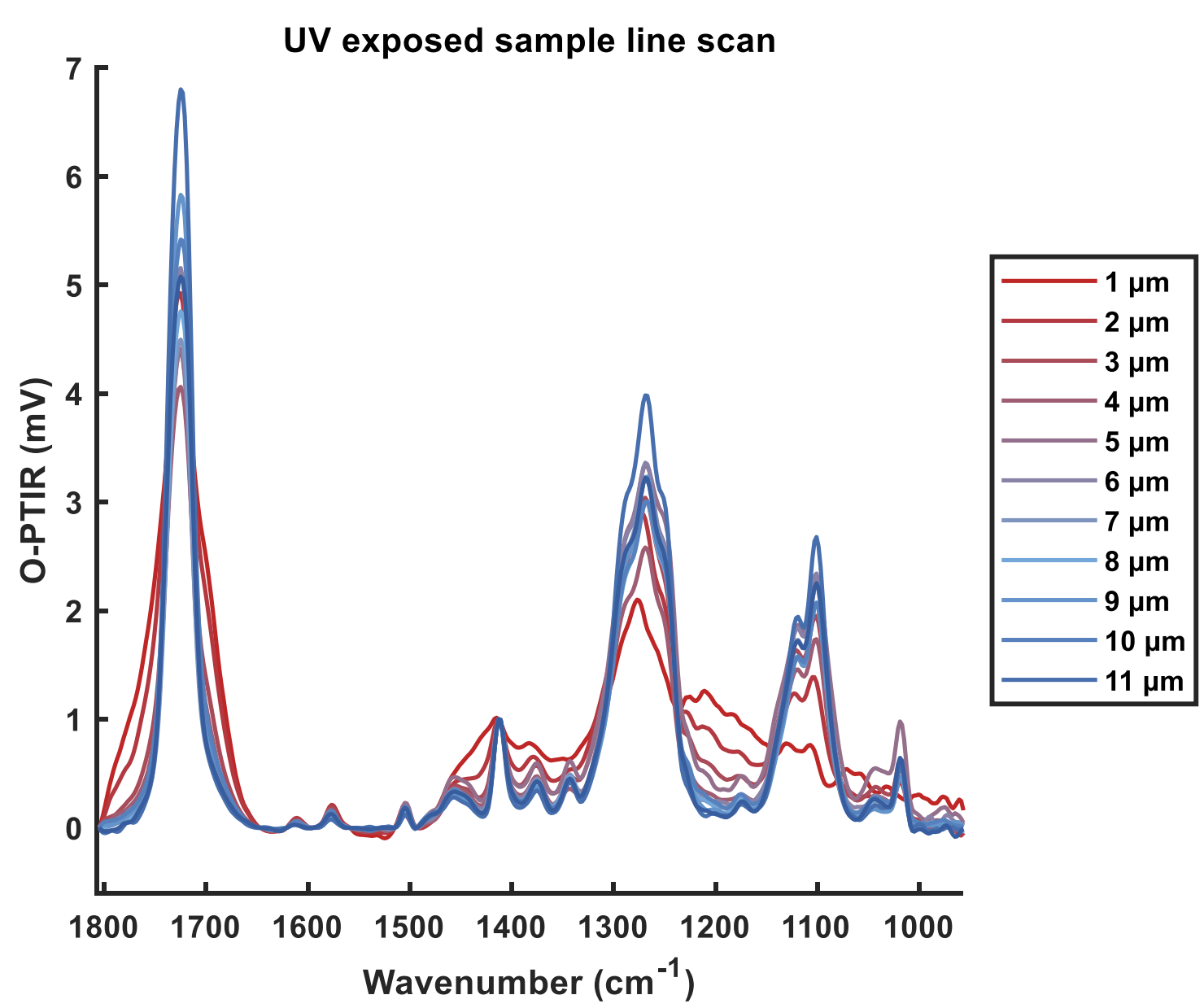

Figure S18. Spectra for the cross-section of 35-day UV-exposed film as a function of depth in µm.

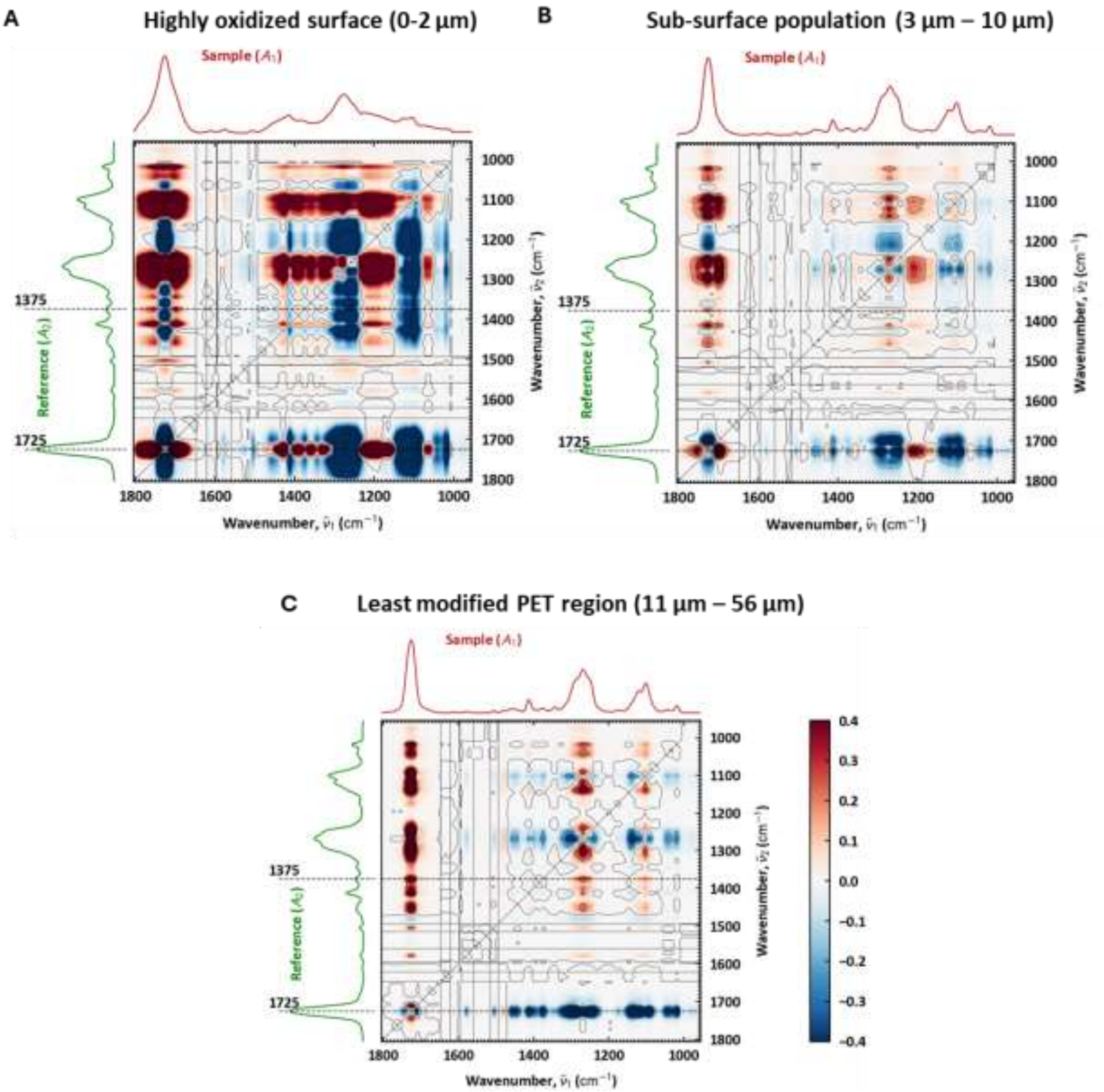

Figure S19. 2T2D correlation maps generated for data in Fig. 5 of the main text from the average spectrum for: A) highly oxidized surface (0 µm - 2 µm), B) sub-surface population (3 µm - 10 µm), and C) least modified PET region (11 µm - 56 µm).

References

1. Cole KC, Ajji A, Pellerin É. New Insights into the Development of Ordered Structure in Poly(ethylene terephthalate). 1. Results from External Reflection Infrared Spectroscopy. *Macromolecules*. 2002;35(3):770-784. doi:10.1021/ma011492i
